# Antagonists of the serotonin receptor 5A target human breast tumor initiating cells

**DOI:** 10.1186/s12885-020-07193-6

**Published:** 2020-08-05

**Authors:** William D. Gwynne, Mirza S. Shakeel, Adele Girgis-Gabardo, Kwang H. Kim, Emily Ford, Anna Dvorkin-Gheva, Craig Aarts, Methvin Isaac, Rima Al-awar, John A. Hassell

**Affiliations:** 1 Department of Biochemistry and Biomedical Sciences, McMaster University, 1280 Main Street West, Hamilton, ON L8S 4L8 Canada; 2 Drug Discovery Group, The Ontario Institute for Cancer Research, 661 University Ave Suite 510, Toronto, ON M5G 0A3 Canada

**Keywords:** Breast cancer, Breast tumorspheres, Breast tumor initiating cells, Serotonin receptor 5A antagonists, Phosphoproteomics

## Abstract

**Background:**

Breast tumor initiating cells (BTIC) are stem-like cells that initiate and sustain tumor growth, and drive disease recurrence. Identifying therapies targeting BTIC has been hindered due primarily to their scarcity in tumors. We previously reported that BTIC frequency ranges between 15% and 50% in multiple mammary tumors of 3 different transgenic mouse models of breast cancer and that this frequency is maintained in tumor cell populations cultured in serum-free, chemically defined media as non-adherent tumorspheres. The latter enabled high-throughput screening of small molecules for their capacity to affect BTIC survival. Antagonists of several serotonin receptors (5-HTRs) were among the hit compounds. The most potent compound we identified, SB-699551, selectively binds to 5-HT5A, a Gα_i/o_ protein coupled receptor (GPCR).

**Methods:**

We evaluated the activity of structurally unrelated selective 5-HT5A antagonists using multiple orthogonal assays of BTIC frequency. Thereafter we used a phosphoproteomic approach to uncover the mechanism of action of SB-699551. To validate the molecular target of the antagonists, we used the CRISPR-Cas9 gene editing technology to conditionally knockout *HTR5A* in a breast tumor cell line.

**Results:**

We found that selective antagonists of 5-HT5A reduced the frequency of tumorsphere initiating cells residing in breast tumor cell lines and those of patient-derived xenografts (PDXs) that we established. The most potent compound among those tested, SB-699551, reduced the frequency of BTIC in ex vivo assays and acted in concert with chemotherapy to shrink human breast tumor xenografts in vivo. Our phosphoproteomic experiments established that exposure of breast tumor cells to SB-699551 elicited signaling changes in the canonical Gα_i/o_-coupled pathway and the phosphoinositide 3-kinase (PI3K)/AKT/mammalian target of rapamycin (mTOR) axis. Moreover, conditional mutation of the *HTR5A* gene resulted in the loss of tumorsphere initiating cells and BTIC thus mimicking the effect of SB-699551.

**Conclusions:**

Our data provide genetic, pharmacological and phosphoproteomic evidence consistent with the on-target activity of SB-699551. The use of such agents in combination with cytotoxic chemotherapy provides a novel therapeutic approach to treat breast cancer.

## Background

Breast tumors comprise a phenotypically heterogeneous tumor cell population [1]. The majority of tumor cells are not tumorigenic, but an infrequent fraction are tumorigenic and possess the stem cell-like properties of self-renewal and differentiation [1]. According to the cancer stem cell model self-renewing BTIC are at the apex of a cellular hierarchy and their differentiation results in non-tumorigenic progeny [2].

Whereas cytotoxic anticancer therapies primarily eradicate non-tumorigenic cells in breast tumors, BTIC are resistant to such therapies [3–7]. Consequently, BTIC frequency increases in tumors after neo-adjuvant chemotherapy [6]. Similar studies have demonstrated that exposure of breast tumor cells to chemotherapy in vitro followed by their transplantation into immune compromised mice (termed ex vivo) also results in an increase in BTIC frequency [7]. The resistance of BTIC to chemotherapy may explain why some breast cancer patients experience a remission after treatment followed by an often-fatal tumor recurrence [8]. Importantly, non-tumorigenic tumor cells can be experimentally induced to become BTIC by initiating an epithelial-to-mesenchymal transition (EMT) [9]. Hence, to improve the durability of remissions therapies must target BTIC and their non-tumorigenic progeny, a potential BTIC reservoir.

Identifying compounds that target BTIC has been challenging due to their scarcity in human tumors [10]. We previously reported that primary tumors arising in multiple transgenic mouse models of breast cancer comprise a high BTIC frequency (15–50% of unpurified tumor cells), which is maintained in vitro by culturing the tumor cells as clonal spheres, which we termed tumorspheres [11]. Other studies have similarly shown that culturing human breast tumor cell lines as tumorspheres results in an increased BTIC frequency by comparison to adherent cells [12, 13]. Hence, the capacity of tumor cells to form tumorspheres is a commonly used in vitro surrogate assay of BTIC frequency [14–16]. To identify inhibitors of BTIC activity we screened 35,000 compounds for their capacity to affect the survival of BTIC-enriched tumorsphere-derived cells using a sensitive assay of cell viability [17].

A fraction of the hits identified in the screen are antagonists of select 5-HTRs and the serotonin reuptake transporter (SERT) [17]. We subsequently demonstrated that structurally unrelated selective antagonists of other serotonin pathway components target mouse [17] and human BTIC [18] using multiple orthogonal assays. In short, antagonists of tryptophan hydroxylase 1 (TPH1), monoamine oxidase A (MAO-A), SERT and each of 5 of the 17 5-HTRs inhibited tumorsphere formation by each of six human breast tumor cell lines independent of the breast cancer subtype that they model [18, 19]. A highly selective 5-HT5A antagonist (SB-699551) was the most potent of all the compounds tested in these assays.

Herein we demonstrate that exposure of human breast tumor cells to several structurally unrelated selective antagonists of 5-HT5A reduced BTIC frequency and that this effect was phenocopied by a CRISPR-Cas9-mediated knockout of *HTR5A.* We used a phosphoproteomic approach to establish that exposure of human breast tumor cells to SB-699551 disrupts signaling via the Gα_i/o_-coupled pathway and the PI3K/AKT/mTOR axis, consistent with antagonism of 5-HT5A. We further showed that treatment of mice in vivo with SB-699551 reduced human breast tumor xenograft growth rate and functioned in concert with docetaxel chemotherapy to shrink the xenografts. Collectively our data provide genetic, pharmacological and phosphoproteomic evidence that 5-HT5A is the likely target of SB-699551 and that selective 5-HT5A antagonists might be developed into a novel class of anticancer agents that can be combined with cytotoxic therapies to shrink established breast tumor xenografts.

## Methods

### Compounds and suppliers

API-2 (2151) was purchased from Tocris Chemicals. Buparlisib (S2247), AZD8055 (S1555) and MK-2206 (S1087) were purchased from Selleckchem. Rapamycin (R5000) was obtained from LC Laboratories. SB-699551 was synthesized by Dalriada Therapeutics Inc. Non-commercially available 5-HT5A antagonists were obtained through a collaboration with the Ontario Institute for Cancer Research.

### Sphere forming assays

Quantitative sphere forming assays were performed as described previously [17, 18]. PrestoBlue (Thermo Fisher Scientific) cell viability assays were performed according to the supplier’s protocol.

### Statistical analyses

Assays were repeated in 2 or more biological experiments with each data point being the average of a minimum of 3 technical replicates. Differences among experimental means were analyzed by analysis of variance (one-way ANOVA). Significant differences between individual means were calculated using Tukey’s test or pairwise t-tests where appropriate. For Kaplan-Meier survival, statistical significance was determined using a log-rank (Mantel-Cox) test. Differences were considered statistically significant if *P* < 0.05. Our in vivo experiment contained enough subjects to detect a 30% difference in tumor volume (mm^3^) at an alpha of 0.05 and a power of 0.80.

### Ethical statement

All experiments utilizing animal models were approved in accordance with the *Animals for Research Act of Ontario* (1980) and the guidelines of the Canadian Council on Animal Care by the McMaster University Animal Research Ethics Board (Animal Utilization Protocol; AUP: 17–09-40). All procedures involving mice were performed within the Canadian Council on Animal Care-approved Central Animal Facility at McMaster. Endpoints outlined in the AUP were strictly adhered to.

### Animal study design

In vivo and ex vivo experiments utilized 6–8-week old female non-obese diabetic/severe combined immune deficient (NOD/SCID) mice obtained from the University Health Network, Toronto. All mice were naïve to prior experimental manipulation. The number of cohorts/ subjects used in each experiment is outlined in the Supplementary Methods. Each mouse was considered one experimental unit and mice were kept in cages of 4–5 mice per cage in pathogen-free housing. Cages were provided constant veterinarian support, food, water, bedding and nesting materials ad libitum*,* and proper light/dark cycling. To minimize experimental bias, mice were randomized into all prospective treatment cages for in vivo preclinical experiments. The injections of tumor cells ex vivo were also blinded.

### Animal experimental procedures

For in vivo and ex vivo studies, viable tumor cells (10,000 or 25,000 tumorsphere derived cells) were transplanted into the #2 mammary fat pad by subcutaneous injection in a 1:1 v/v solution of phosphate buffered saline (PBS) and Matrigel (Corning). To aid precise injections and minimize injection number, mice were anaesthetized with isoflurane gas. SB-699551 and docetaxel were administered as 100 μL intraperitoneal injections (each morning; alternating sites with a 28-gauge insulin syringe) in a vehicle consisting of 10% DMSO, 9% Kolliphor EL (Sigma) and 81% PBS. Xenograft incidence was monitored by daily palpation and tumor volume was determined using digital calipers [20]. All mice tolerated the treatment protocols and remained active throughout the study (Supplementary Figure 5). After mice reached experimental endpoint as outlined in the AUP (vehicle mice larger tumor volume), they were euthanized by exposure to carbon dioxide followed by cervical dislocation. At endpoint tumor specimens were resected and tumor mass was determined using a digital scale (sensitivity 0.1 mg). Formalin-fixed paraffin-embedded (FFPE) sections of the xenografts were prepared for analyses.

### Molecular cloning

We used ultramer primers (IDT) comprising unique sgRNA sequences to PCR-amplify the regions of lenti-multi-guide that encode the hU6 promoter and the sgRNA scaffold, yielding multi-guide expression cassettes [21]. After BsmBI-mediated digestion, the cassettes were cloned into lenti-multi-guide in a single ligation reaction. sgRNA sequences were generated using CRISPR Era (http://crispr-era.stanford.edu/). Constructs were sequence-verified by Sanger sequencing.

### Lentivirus production

Five million HEK293T cells were seeded into 10-cm plates and transfected with 15 μg of the transfer plasmid, 10 μg of psPAX2 and 5 μg of pMD2.G using 75 μL Lipofectamine 2000 (Thermo Fisher Scientific). Supernatants were collected and concentrated using LentiX (Takara). Virus titers were determined in MCF-7 cells by colony forming assays.

### *HTR5A* inducible knockout (iKO)

MCF-7 cells were infected with Lenti-iCas-Neo [21] at a multiplicity of infection (MOI) of 0.3. After selection in G418-containing media, clonal cell lines were isolated and characterized for their capacity to express Cas9-GFP upon induction (inducible Cas9; iCas9) with doxycycline (dox). iCas9 cell lines were infected with Lenti-multi-guide [21] plasmids (MOI 0.3). After puromycin selection, clonal *HTR5A* iKO lines were isolated.

### Cell lines

All commercially available cell lines (HCC1954, MDA-MB-157 and MCF-7) were obtained from the American Type Culture Collection (ATCC). ATCC uses STR profiling to validate cell lines. Cell lines were expanded upon arrival and stored in liquid nitrogen as early passage stocks. All experiments herein used these early passage stocks. Patient-derived cell lines were isolated from our previously established patient-derived xenografts (PDXs) [18].

### Next-generation sequencing

To characterize clonal iKO lines, cells were grown for 48 h in DMEM (10% FBS) with 1 μg/mL doxycycline (dox) or its vehicle (H_2_O). To generate crude genomic DNA (gDNA) lysates, of approximately 100,000 iKO cells were lysed in Food Safety buffer (Thermo Fisher Scientific) diluted 1:3 v/v in H_2_O containing Proteinase K. The sgRNA-binding region of gDNA was amplified by PCR using Illumina adapter-containing primers that bind approximately 75 base pairs upstream and downstream of the respective pan adjacent motif. Thereafter, Illumina indices were added to the ends of each amplicon with 3–4 additional PCR cycles. The resulting samples were sequenced using MiSeq. FastQ files were aligned to the consensus *HTR5A* sequence using Geneious 8.0 to determine the INDEL frequency. The translational consequence of INDELs was determined by filtering and trimming reads using Geneious BBDUK, with an average read quality filter of at least 20 and then translating the entire amplicon. Reads that maintained the C-terminal amino acid sequence of each amplicon were determined to be in frame.

### iKO assays

We seeded T25 flasks with 300,000 viable tumorsphere-derived cells on day 0, and then propagated the tumorspheres in either dox-free or dox-containing, chemically defined media for 3 sequential passages every 4 days. At each passage a 200 μL aliquot of cells was plated in the wells of 96-well dishes in triplicate to determine the number of tumorspheres that arose after each passage. For ex vivo assays we isolated viable tumor cells after the third passage and transplanted each of 10,000 or 25,000 viable tumor cells into cohorts of 4 NOD/SCID mice.

### Proteomic profiler Array

The Proteomic Profiler Array (PPA; R&D Systems) analyses were performed following the instructions provided by the manufacturer with 600 μg of protein lysate. The membranes were exposed to X-Ray films (Thermo Scientific), which were subsequently scanned using a Hewlett-Packard scanner (Supplementary Figure 2; Supplementary Table 1). Mean pixel densities of phosphoproteins on the film were determined using Image Studio.

### Western blotting

Protein lysates were isolated from breast tumor cell lines by solubilization in RIPA buffer. Protein concentrations were estimated using the Bradford assay (Bio-Rad) and 25 μg of each sample was resolved in denaturing conditions in a 4–12% gradient polyacrylamide gel. Proteins were transferred onto polyvinylidene difluoride (PVDF) membranes using the iBlot (Thermo Fisher Scientific). The membrane was washed in PBS with 0.1% Tween-20 (PBS-T) and then blocked for one hour at room temperature in PBS-T containing 5% bovine serum albumin. Primary antibodies for pAKT(S473) (#4060), pAKT (T308) (#13038), AKT (#2938), pPRAS40 (#2997), PRAS40 (#2691), pCREB (#9198), CREB (#9197), pFOXO1 (#9461) and FOXO1 (#2880) were purchased from Cell Signaling Technologies. Phosphoprotein abundance was determined by normalizing phosphoprotein pixel density to that of the total protein.

### Staining of xenograft sections

HCC1954 tumor xenografts were resected from mice at endpoint and fixed in 10% formalin for 48 h. Fixed specimens were embedded in paraffin and then sectioned. Sections were dewaxed using xylenes and rinsed in 95% ethanol. Dewaxed sections were stained using hematoxylin and eosin for histological analysis via light microscopy. Sections were also stained with the Click-IT TUNEL assay (ThermoFisher) and imaged under the FITC channel. Scale bars were introduced using ImageJ software.

## Results

### Structurally unrelated 5-HT5A selective antagonists reduce the frequency of tumorsphere initiating cells in breast cancer cell lines and those derived from human patient-derived tumors

**Fig. 1:**
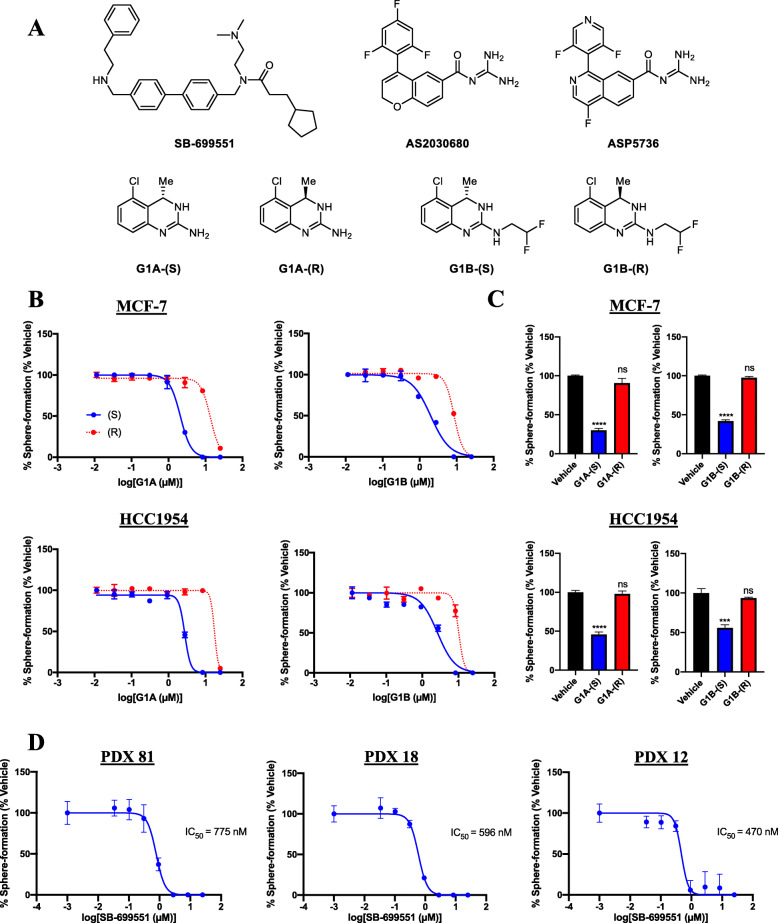
Selective antagonists of 5-HT5A inhibit tumorsphere formation by human breast tumor cells. **a** Panel of structurally distinct selective antagonists of 5-HT5A. **b** IC_50_ curves (Graphpad Prism 7.0) generated from non-linear regression of sphere forming assays with enantiopure guanidine-like 5-HT5A antagonists. The blue curves show the effect of increasing doses of the (S) isomers on sphere formation whereas the red curves show the effect of the (R) isomers. Each point indicates the mean sphere forming efficiency +/− the standard error of the mean (SEM). **c** Bar graphs showing that the frequency of sphere initiating cells was unaffected by treatment with G1A-(R) and G1B-(R) (red) at the approximate IC_50_ concentrations (2.7 μM) of their (S)-enantiomers (blue). Statistical significance was determined by one-way ANOVA and post-hoc Tukey’s tests [**** *p* < 0.0001; *** *p* = 0.006; ns *p* > 0.05]. **d** IC_50_ curves of sphere forming assays using PDX derived cells lines exposed to increasing concentrations of SB-699551. A 0.1 nM concentration of each compound was used to represent the vehicle allowing for accurate IC_50_ calculations

We reasoned that if SB-699551 targets tumorsphere initiating cells by inhibiting 5-HT5A, then structurally unrelated selective 5-HT5A antagonists ought to reproduce this finding. To address the latter, we procured the synthesis of a panel of selective 5-HT5A antagonists, which are not commercially available (Fig. 1a). Three compounds (SB-699551, AS2030680 and ASP5736) are highly selective for 5-HT5A over all other 5-HTR subtypes [22, 23]. The (S)-isomers of the guanidine-type antagonists (G1A-(S) and G1B-(S)) display a 4- and 7-fold selectivity for 5-HT5A over 5-HT7 and are more selective for these two 5-HTRs by comparison to all other 5-HTRs [24]. We tested the capacity of each compound to affect the frequency of tumorsphere initiating cells resident in the HCC1954 (human epidermal growth factor receptor 2 (HER2)-overexpressing; basal molecular subtype) and MCF-7 (estrogen receptor positive (ER^+^); luminal molecular subtype) human breast tumor cell lines. We also used PrestoBlue assays in parallel to learn whether the compounds affected the viability of the tumor cells.

SB-699551, AS2030680, G1A-(S) and G1B-(S) all inhibited tumorsphere formation with half-maximal inhibitory concentrations (IC_50_) ranging from 0.3 to 3.0 μM (Table 1), with SB-699551 exhibiting the highest potency. G1A-(S) and G1B-(S) were more potent in each of the assays by comparison to their (R)-configured stereoisomers, which have a much lower affinity for 5-HT5A [24] (Fig. 1b; Supplementary Figure 1). Indeed, G1A-(R) and G1B-(R) were inactive at the IC_50_ concentrations of their (S)-enantiomers (Fig. 1c). The enantiomeric selectivity of the (S) isomers is consistent with their binding affinities for 5-HT5A providing pharmacological evidence for their on-target activity [24]. Interestingly ASP5763, which is structurally related to ASP2030680, was inactive up to the highest concentration tested in both assays.

**Table 1:** Selective antagonists of 5-HT5A inhibit sphere formation by human breast tumor cells

Compound	Cell Line	IC50 (μM)	
		Sphere Formation	PrestoBlue Reduction
SB-699551	HCC1954	0.3	0.2
	MCF-7	0.2	0.2
G1A-(S)	HCC1954	2.5	6.7
	MCF-7	2.5	3.0
G1A-(R)	HCC1954	16.4	14.2
	MCF-7	11.0	21.9
G1B-(S)	HCC1954	3.0	3.8
	MCF-7	2.2	1.3
G1B-(R)	HCC1954	11.3	11.8
	MCF-7	11.4	14.1
AS2030680	HCC1954	1.8	3.9
	MCF-7	2.0	5.6
ASP5736	HCC1954	> 25.0	> 25.0
	MCF-7	> 25.0	> 25.0

Tumorsphere derived cells were exposed to each of 7 concentrations of each compound or its vehicle and then incubated for 72 h prior to sphere forming and PrestoBlue reduction assays. The IC_50_ values (in μM) were calculated by nonlinear regression of dose-response curves using GraphPad Prism 7.0. A minimum of three technical replicates were included for each concentration of the compounds

Because our initial screening of selective 5-HT5A antagonists used human breast tumor cell lines, we wondered whether SB-699551 would similarly affect the frequency of tumorsphere initiating cells in patient-derived breast tumor cell lines. To investigate the latter, we used breast tumor cells from three PDXs that we had established [18], which comprise distinct molecular subtypes of breast cancer. PDX 81 was derived from an ER^+^ breast tumor and is of the luminal molecular subtype, whereas PDX 18 and PDX 12 were derived from triple-negative breast tumors and are of the basal molecular subtype. All three PDX-derived cell lines responded similarly to treatment with SB-699551 (Fig. 1d), suggesting that selective antagonists of 5-HT5A eliminate tumorsphere initiating cells in breast tumor cell lines and PDX-derived cell lines independent of their subtype.

### SB-699551 affects tumorsphere formation by an irreversible mechanism and targets BTIC

**Fig. 2:**
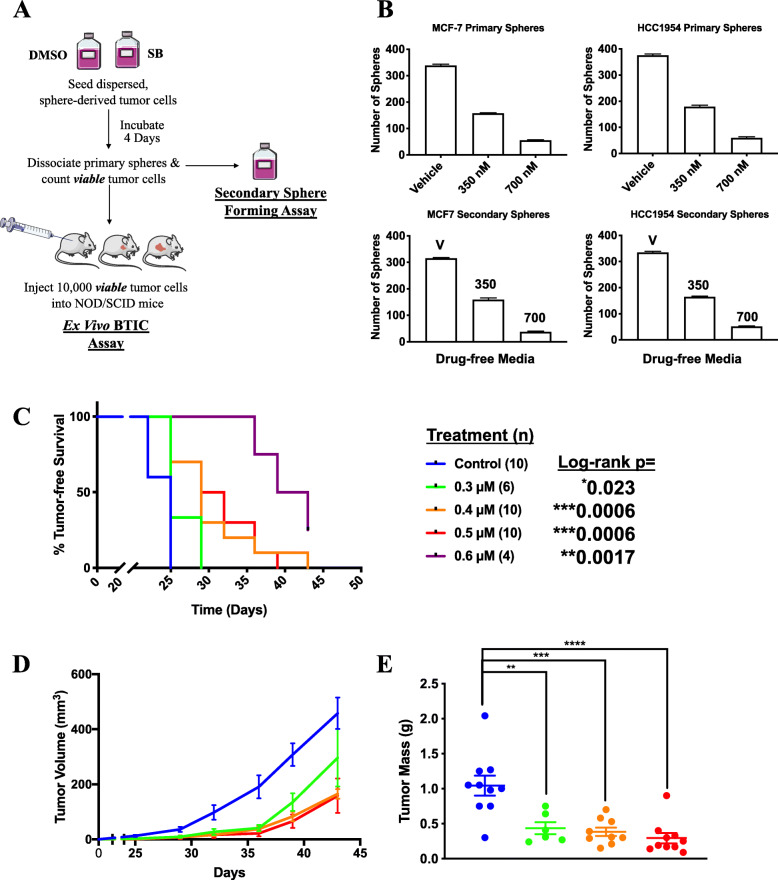
SB-699551 affects tumorsphere formation by an irreversible mechanism and targets BTIC. **a** Schematic of a secondary sphere forming assay and an ex vivo assay of BTIC activity. **b** The number of tumorspheres that formed in the primary sphere forming assay after exposure to the vehicle or the IC_50_ and IC_90_ concentration of SB-699551, and the number of tumorspheres that arose in the secondary sphere forming assay in SB-699551-free media. **c** Kaplan-Meier survival analysis of mice that were injected with an equal number of viable HCC1954 tumor cells exposed to the vehicle or to various concentrations of SB-699551. Statistical significance was determined by Kaplan-Meier survival analysis and the log-rank test. **d** Growth curves of the xenografts after transplantation of an equal number of viable tumor cells exposed to the vehicle or to the various concentrations of SB-699551. Each point represents the mean volume of xenografts that arose after transplantation +/− the SEM. **e** Xenograft mass at endpoint in grams (g). Statistical significance was determined using one-way ANOVA and post-hoc Tukey’s tests. [**** p < 0.0001; *** *p* = 0.0002; ** *p* = 0.0025]. The three xenografts arising from mice engrafted with 0.6 μM-treated tumor cells were tiny nodules and were thus excluded from volume and weight analyses

We have previously reported that compounds that irreversibly inhibit the activity of tumorsphere initiating cells and BTIC do so by initiating irreversible cellular processes such as apoptosis and/or differentiation [16]. If SB-699551 inhibits tumorsphere formation by an irreversible mechanism, then their frequency ought to be reduced in secondary sphere forming assays (Fig. 2a) [16–18, 25]. In short, MCF-7 and HCC1954 tumorsphere-derived cells were exposed to the vehicle (0.01% dimethyl sulfoxide; DMSO) or to each of two concentrations of SB-699551. The tumorspheres that arose were dissociated and an equal number of viable cells was reseeded into compound-free media and the resulting number of spheres determined. Whereas cells exposed to the vehicle formed tumorspheres in the secondary assay at an identical frequency (~ 5%) as that in the primary assay, the frequency of tumorsphere initiating cells was reduced after exposure to SB-699551 in a dose-dependent fashion (Fig. 2b) demonstrating that the antagonist targeted the tumorsphere initiating cells by an irreversible mechanism.

To ensure that BTIC frequency per se was affected by exposure to SB-699551, we assessed its capacity to affect tumor initiation using ex vivo assays [7] (Fig. 2a). We used the HCC1954 cell line for these experiments because we previously demonstrated that it forms xenografts rapidly and synchronously [18]. In short, we exposed HCC1954 tumorsphere derived cells to media containing the vehicle or to each of 4 concentrations of SB-699551. After a 72-h incubation period the tumorspheres that formed were dissociated and viable dispersed tumor cells were transplanted into the #2 mammary fat pad of 8-week old female NOD/SCID mice.

Palpable tumor xenografts first appeared in the cohort of mice transplanted with vehicle-treated tumorsphere-derived cells on day 22, and by day 25 all mice in this cohort had a palpable tumor (Fig. 2c). Xenograft formation was statistically significantly delayed in cohorts engrafted with tumor cells exposed to SB-699551 in a dose-dependent fashion. Consistent with these findings, xenografts arising from SB-699551-treated tumor cells exhibited a reduced growth rate (Fig. 2d) and mass (Fig. 2e) at endpoint by comparison to vehicle-treated controls. We and others have previously shown that tumor latency, growth rate and volume at endpoint are directly correlated with the BTIC frequency of the transplanted tumor cells [11, 26]. These data demonstrate that SB-699551 targets BTIC in HCC1954 tumorsphere-derived cell populations.

### Treatment of breast tumor cell lines with SB-699551 affects signaling pathways downstream of 5-HT5A

5-HT5A is a G-protein coupled receptor (GPCR) and signals via Gα_i/o_, which inhibits the activity of adenylate cyclases (AC) thereby preventing the accumulation of intracellular cyclic-AMP (cAMP) [23]. Serotonin-mediated signaling is terminated by GPCR kinases that phosphorylate serine and threonine residues within intracellular regions of 5-HTRs, which serve as docking sites for β-arrestins that facilitate receptor internalization [27]. Notably, β-arrestins act as scaffolds for kinases such as SRC, AKT, phosphoinositide 3-kinase (PI3K), extracellular signal-regulated kinases (ERK1/2) and receptor tyrosine kinases [28–31]. The Gβ/γ subunit of heterotrimeric G-proteins also couples to signaling pathways like AKT by interacting with PI3K [32].

**Fig. 3:**
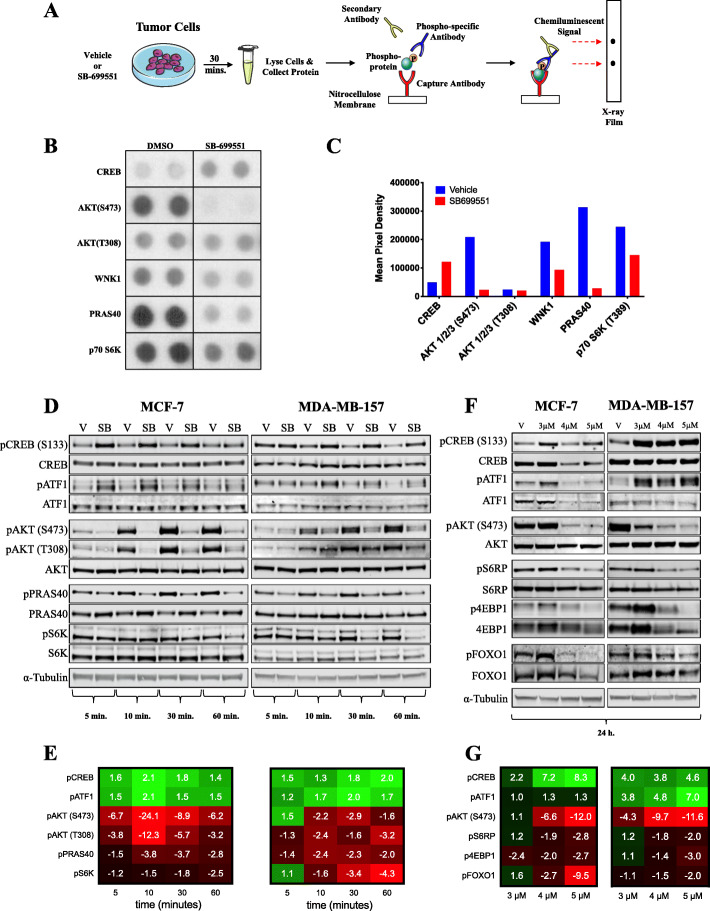
SB-699551 signals via canonical Gα_i/o_ coupling and through the PI3K/AKT/mTOR pathway. **a** Schematic outlining the procedure followed for the PPA. **b** The dot blots from X-ray film that correspond to the most notable phosphoproteins whose abundance changed after treatment with SB-699551. The full-length X-ray film is shown in Supplementary Figure 2. **c** Mean pixel densities of candidate phosphoproteins in MCF-7 tumor cells grown under each condition. **d** Western blotting validation of temporal phosphoproteomic changes in the MCF-7 and MDA-MB-157 breast tumor cell lines for up to a 60-min exposure to the vehicle (0.1% DMSO) or SB-699551 (SB; 4 μM). Phosphoprotein abundance (pixel density) was calculated by the ratio of phosphoprotein to that of the total protein, each normalized to an alpha (α)-Tubulin loading control. **e** Heatmap showing fold increase (green) or decrease (red) of phosphoprotein abundance in SB-699551-treated cells relative to those treated with the vehicle. **f** Western blotting validation of phosphoproteomic changes in MCF-7 and MDA-MB-157 breast tumor cells lines after a 24-h exposure to the vehicle (V) or SB-699551 (3 μM, 4 μM or 5 μM). **g** Relative phosphoprotein abundance after a 24-h exposure of MCF-7 and MDA-MB-157 cells to SB-699551. Western blot frames (outlined in black) were cropped; full-length blots are shown in Supplementary Figure 8. Blots were imaged using the LI-COR Biosciences Odyssey Platform and analysed using ImageStudio software

To determine whether SB-699551 affects BTIC survival by interfering with signaling downstream of 5-HT5A, we sought to identify the signaling pathways that might be affected in breast tumor cell lines after treatment with the compound. To pursue this objective, we used the PPA, which measures the phosphorylation status of 43 intracellular proteins using phospho-specific antibodies in a sandwich ELISA format. Protein lysates were isolated from MCF-7 tumor cells treated with the vehicle (0.1% DMSO) or SB-699551 (4 μM) for 30 min and analyzed using the PPA (Fig. 3a; Supplementary Figure 2). The phosphoproteins whose abundance were most affected by treatment with SB-699551 are shown in Fig. 3b-c.

Treatment of MCF-7 cells with SB-699551 resulted in the increased phosphorylation of the cAMP-response element binding protein (CREB), which is consistent with inhibition of the 5-HT5A-coupled Gα_i/o_ pathway, resulting in the increased activity of protein kinase A (PKA) and the phosphorylation of CREB. We also found that SB-699551 treatment reduced the phosphorylation of AKT at serine residue 473 (S473) and that of two of its downstream effectors, namely with no lysine kinase 1 (WNK1) and the proline-rich AKT substrate (PRAS40). Interestingly, unphosphorylated PRAS40 inhibits the activity of the mammalian target of rapamycin (mTOR). Consistent with the latter, SB-699551 treated tumor cells exhibited reduced phosphorylation of the p70-S6 kinase 1 (S6K), a direct target of mTOR.

To validate the results of the PPA we used Western blotting with antibodies capable of binding to the phosphorylated or all forms of each protein investigated (Fig. 3d-e). To determine the temporal changes in protein phosphorylation we isolated cell lysates from breast tumor cells that were treated with SB-699551 for 5, 10, 30 or 60 min. We included a vehicle control at each time point to account for any effect of media changes on protein phosphorylation. We selected the MCF-7 cell line to validate the PPA and expanded our analysis to include the TNBC cell line of the basal molecular subtype, MDA-MB-157.

We found that treatment of the breast tumor cells with SB-699551 increased the phosphorylation of CREB in the MCF-7 cell line by a similar magnitude as that observed in the PPA. Interestingly, the antibodies that bind total CREB and pCREB also bind to cAMP-dependent transcription factor (ATF1), which like CREB is a member of the ATF family of transcription factors and a substrate of PKA. The phosphorylation of ATF1 also increased in SB-699551-treated MCF-7 and MDA-MB-157 cells and followed a similar temporal pattern as that of CREB phosphorylation. These observations offer additional evidence that SB-699551 acts by inhibiting Gα_i/o_-coupled signaling resulting in the accumulation of cAMP and activation of PKA.

In agreement with the PPA data we observed a substantial decrease in the phosphorylation of pAKT (S473) occurring as early as 5 min after treatment of both cell lines with SB-699551. Whereas differences in the phosphorylation of AKT at threonine residue 308 (T308) were not apparent in the PPA, we detected a noticeable decrease using Western blotting. The latter may reflect differences in the binding affinities of the primary antibodies used in each assay format. Interestingly, the phosphorylation of AKT increased in the vehicle treated cells in a time-dependent fashion, likely due to the addition of fresh serum-containing media, which contains growth factors that activate AKT signaling [33].

We also validated alterations in mTOR signaling by examining the phosphorylation of PRAS40 and S6K. Whereas the phosphorylation of PRAS40 was reduced as early as 5 min after treatment with SB-699551, that of pS6K was not affected until 30–60-min. To account for changes in the phosphorylation of mTOR targets that may require a longer incubation period, we isolated lysates from MCF-7 and MDA-MB-157 cells treated with the vehicle or different concentration of SB-699551 (3, 4 or 5 μM) for 24 h. Interestingly, the decreased phosphorylation of pAKT, pCREB and pATF1 observed previously was maintained after 24 h of SB-699551 treatment (Fig. 3f-g).

We examined the phosphorylation level of the S6 ribosomal protein (S6RP) a direct target of S6K. We found that its abundance was markedly reduced by SB-699551 in both cell lines. We similarly examined the abundance of the 4E-binding protein 1 (4EBP1), which is a direct substrate of mTOR. We found that the abundance of both p4EBP1 and total 4EBP1 were markedly reduced by treatment with SB-699551. The latter likely occurred because hypo-phosphorylated 4EBP1 is prone to ubiquitination and degradation [34]. Taken together these data show that SB-699551 affects intracellular signaling via the PI3K/AKT/mTOR signaling axis.

AKT mediates cell survival by phosphorylating the forkhead box protein 1 (FOXO1) transcription factor [35]. Phosphorylated FOXO1 (pFOXO1) is sequestered in the cytosol preventing it from effecting the transcription of pro-apoptotic genes such as *BIM*, a member of the *BCL2* gene family. We suspected that SB-699551 may affect the survival of BTIC by AKT mediated inhibition of FOXO1 activity resulting in their apoptosis. We found that the abundance of pFOXO1 was markedly reduced 24 h after treatment of the cells with SB-699551. Consequently, the ratio between active (unphosphorylated) FOXO1 and inactive pFOXO1 was increased in breast tumor cells after treatment with SB-699551. Hence, inhibition of 5-HT5A by SB-699551 may affect the activity of BTIC in part by blocking the capacity for AKT to prevent apoptosis mediated by FOXO1.

### Selective antagonists of kinases downstream of 5-HT5A phenocopy the effect of SB-699551

**Fig. 4:**
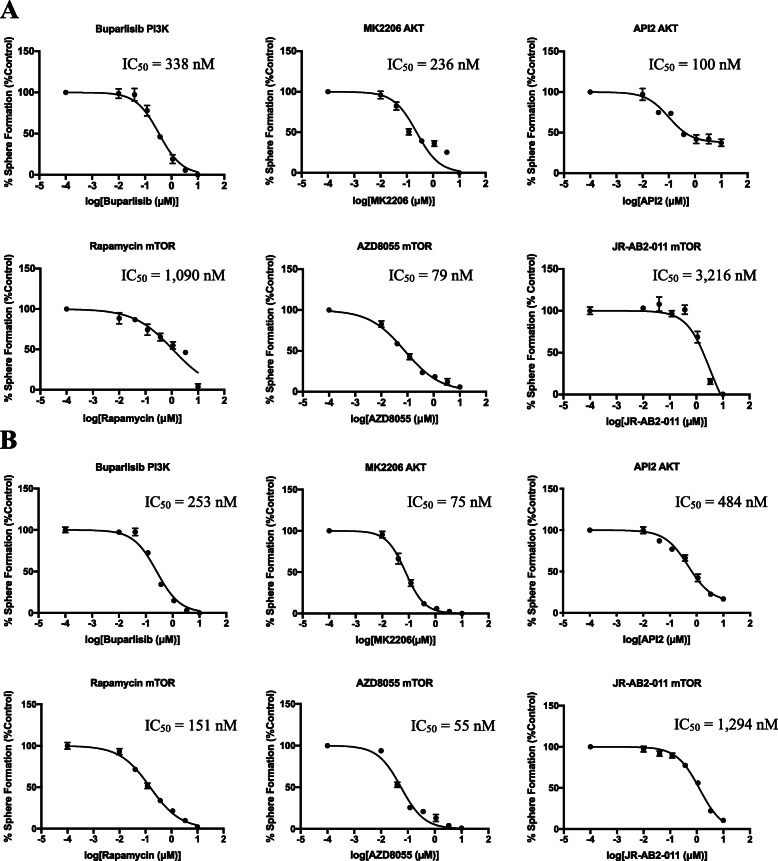
Selective antagonists of the PI3K/AKT/mTOR pathway phenocopy the effect of SB-699551 in sphere forming assays. **a-b** IC_50_ curves of quantitative sphere forming assays in **(a)** MDA-MB-157 and **(b)** MCF-7 tumor cells. Each point indicates the mean sphere forming efficiency of tumor cells at each concentration of each inhibitor +/− the SEM. IC_50_ values (in nM) are listed in the upper righthand corner of each panel

If SB-699551 mediated inhibition of BTIC frequency results from compromising PI3K/AKT/mTOR signaling, then selective inhibitors of these kinases ought to phenocopy the effect of SB-699551. To investigate the latter, we tested the capacity of small molecule antagonists selective for each kinase to affect tumorsphere formation. Included in these experiments were the PI3K inhibitor Buparlisib; two AKT inhibitors MK2206 and API2; the mTOR complex 1 inhibitor rapamycin; the mTOR complex 2 inhibitor JR-AB2–011; and the AZD8055 rapalog, which inhibits both mTOR complexes 1 and 2. We tested the capacity of each compound to inhibit tumorsphere formation in vitro by MDA-MB-157 (Fig. 4a) and MCF-7 (Fig. 4b) breast tumor cells.

Each of the kinase inhibitors reduced the frequency of tumorsphere initiating cells resident in the MCF-7 and MDA-MB-157 breast tumor cell lines with similar IC_50_ values ranging between 50 nM to 3 μM. The latter finding demonstrates that selective antagonists of PI3K, AKT and mTOR phenocopy the effect of SB-699551 in sphere forming assays. Hence, the activity of the kinases downstream of 5-HT5A that we identified using phosphoproteomic analyses are required for the activity of tumorsphere initiating cells.

### Conditional knockout of HTR5A phenocopies the effect of SB-699551

If selective antagonists of 5-HT5A elicit their effects through an on-target mechanism, then genetic loss of *HTR5A* should phenocopy their effects in functional assays. To investigate the latter, we used the CRISPR-Cas9 gene editing technology to conditionally mutate *HTR5A* in MCF-7 breast tumor cells. Because SB-699551 reduces the frequency of tumorsphere initiating cells in breast tumor cell lines [18] we elected to use an inducible system to achieve this objective.

**Fig. 5:**
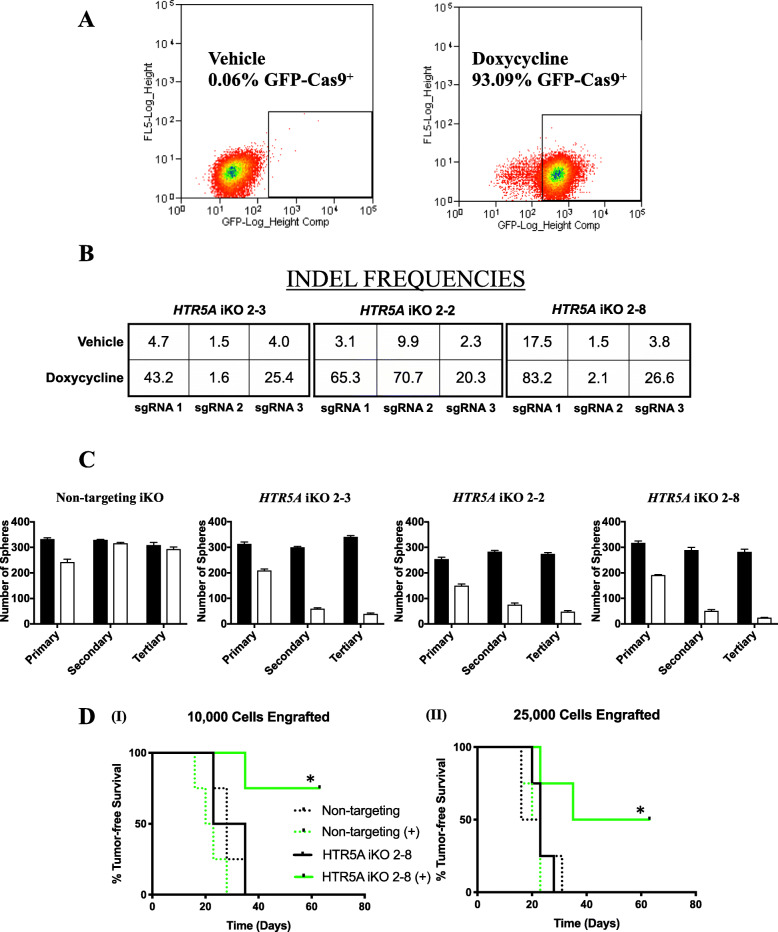
Inducible knockout of *HTR5A* affects tumorsphere formation and targets BTIC. **a** FACS scatter plot showing the frequency of Cas9-GFP^+^ tumor cells in an MCF-7 iCas9 cell line grown in dox-containing or dox-free media for 48 h. **b** Percentage INDEL frequency at each sgRNA-binding locus of *HTR5A* was obtained by next-generation sequencing of genomic DNA isolated from 3 MCF-7 *HTR5A* iKO clones grown in dox-containing media or the vehicle for 48 h. **c** The number of tumorspheres formed in media containing the vehicle (black bars) or dox (white bars) for each cell line was determined over 3 successive passages. Bar graphs display the mean number of tumorspheres +/− the SEM. **d** Ex vivo assays demonstrate that induction of Cas9 expression in the *HTR5A* iKO 2–8 cell line (solid lines) delays the onset of tumor xenografts (compare uninduced (black) with dox-induced (green)), whereas induction of Cas9 expression has no effect on the appearance of tumor xenografts in the NT iKO cell line (dotted lines). Statistical significance was determined using Kaplan-Meier survival analysis and the log-rank test [(I) * *p* = 0.0047; (II) * *p* = 0.01]

In short we isolated clonal MCF-7 cell lines capable of expressing a self-cleaving Cas9-GFP fusion protein under the control of a dox-inducible promoter (Fig. 5a) and constitutively expressing 3 sgRNA complementary to both exons of the *HTR5A* gene [21]. We also generated an analogous MCF-7 clonal cell line that expresses 3 non-targeting (NT) sgRNAs that are not complementary to sequences in the human genome as a control. Three clonal MCF-7 cell lines (2-2, 2-3 and 2-8), displayed tight regulation of dox-induced Cas9 mediated insertions/deletions (INDELS) in *HTR5A* (inducible knockout; iKO) (Fig. 5b), as established by next-generation sequencing of genomic DNA. We translated INDEL containing reads in silico to establish the consequences of Cas9-mediated mutagenesis of exons during a 48-h period of exposure to dox (Supplementary Figure 7A). We found that 66–84% of these mutations shifted the reading frame, whereas 16–34% maintained the reading frame, resulting in amino acid insertions or deletions. The latter ranged in size from a single amino acid residue to as many as 33 amino acids (Supplementary Figure 7B-C).

We used the genetically modified MCF-7 clonal cell lines to determine whether the loss of 5-HT5A activity affected their capacity to form tumorspheres. If 5-HT5A activity is required for the survival of the tumorsphere initiating cells, then their frequency would be expected to decline after each successive passage of the tumorspheres in dox-containing media by comparison to those propagated in dox-free media. The latter expectation was realized for all 3 clonal cell lines that inducibly express sgRNAs targeting *HTR5A*, but not in those expressing the NT sgRNAs (Fig. 5c).

To ensure that BTIC frequency per se was affected by loss of 5-HT5A activity, we performed ex vivo *assays* with the MCF-7 NT iKO and *HTR5A* iKO 2–8 cell line. In short, we repeated the experiment outlined above and at the end of the last passage tumorspheres were dissociated and each of 10,000 or 25,000 viable cells were transplanted into the #2 fat pad of 4 immune-compromised NOD/SCID mice. The time to occurrence of tumor xenografts was monitored over the course of 8 weeks. The addition of dox to the media did not affect the time to appearance of the tumor xenografts seeded by the NT cell line (Fig. 5d). By contrast, xenografts seeded by *HTR5A* iKO 2–8 cells cultured in presence of dox were delayed in their occurrence by comparison to those seeded by the cells propagated in the absence of dox. These findings demonstrate that conditional mutation of *HTR5A* results in a reduction in the frequency of BTIC thus phenocopying the effect of SB-699551 in the same functional assay.

### Treatment with SB-699551 affects the growth of breast tumor xenografts

Prior to initiating a preclinical study with SB-699551, we assessed its potential toxicity in vitro and in vivo in mammary epithelial cells. Our previously published compound screen [17] used BTIC-enriched tumorspheres from the MMTV-*Neu* transgenic model of breast cancer [11]. We isolated mammary epithelial cells from wildtype mice of the same strain (FVB) and propagated them as mammospheres, providing us with a biologically matched control to determine whether SB-699551 preferentially targets tumorsphere initiating cells by comparison to those that initiate mammosphere formation (Supplementary Figure 3). These experiments established a modest but statistically significant therapeutic window, demonstrating that at the IC_50_ of SB-699551 in tumorspheres, the compound had no effect on the frequency of mammosphere initiating cells.

**Fig. 6:**
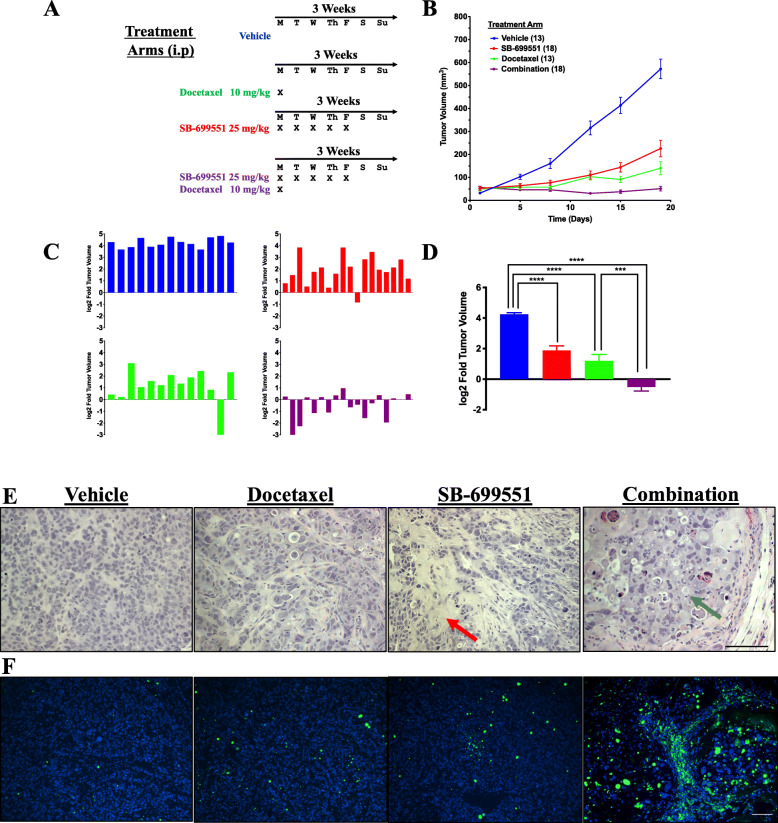
Treatment with SB-699551 inhibits the growth of human breast tumor xenografts in vivo. **a** Twenty-five thousand HCC1954 breast tumor cells were injected into the mammary fat pad of NOD/SCID mice. After four weeks mice bearing tumor xenografts that had reached a volume between 50 and 100 mm^3^ were randomized into four treatment arms comprising either 13 or 18 mice. **b** Tumor volume was monitored every four days with digital calipers and the mean xenograft volume +/− the SEM was plotted over the course of 18 days. **c** The ratio of the final tumor xenograft volume at endpoint compared to their starting volume was calculated for each individual xenograft-bearing mouse. **d** The mean difference in tumor growth was analyzed for statistical significance using a one-way ANOVA and post-hoc Tukey’s tests [**** *p* < 0.0001; *** *p* = 0.0005]. **e** Representative sections of xenografts stained with hematoxylin and eosin. The red arrows indicate areas of the xenografts with low tumor cell density and the green arrows indicate tumor cells with shrunken, pyknotic nuclei. **f** TUNEL staining of xenograft sections. The DAPI channel (blue) marks nuclei and the FITC channel (green) marks the FITC-labeled nicked DNA of apoptotic cells. The scale bar represents 100 μm

In advance of testing SB-699551 in preclinical experiments we established its maximum tolerated dose in combination with 10 mg/kg of docetaxel (Supplementary Table 2). We found that 25 mg/kg was well tolerated in combination with docetaxel. We then established tumor xenografts by transplanting 25,000 tumorsphere-derived HCC1954 cells into the #2 mammary fat-pad of NOD/SCID mice. We chose this cell line because we have previously demonstrated that it rapidly and synchronously forms xenografts in NOD/SCID mice [18]. After 4 weeks, mice bearing tumor xenografts with an approximate volume of 50 mm^3^ were randomized into 4 treatment arms after which we initiated a 3-week treatment regimen as outlined in Fig. 6a. Tumor volume in each mouse was determined every 3–4 days using digital calipers for the duration of the treatment regimen.

The growth rates of the xenografts in all compound treated cohorts were reduced by comparison to those treated with the vehicle (Fig. 6b). To assess the effect of each treatment on individual tumors and to correct for any variation in their starting volume, we compared the volume of each tumor xenograft at endpoint to its initial volume (Fig. 6c). All the xenografts from the vehicle-treated mice grew to the greatest extent throughout the treatment cycle and at endpoint the mean log2 fold change in volume (log2FC) was 4.2 (Fig. 6d). In the docetaxel- or SB-699551-treated mice, all but one xenograft in each of the 2 cohorts grew throughout the course of treatment. The mean log2FCs were 1.9 and 1.2 for the SB-699551- and docetaxel-treated cohorts, respectively. By contrast, half of the xenografts in mice receiving a combination of both agents shrank during the treatment period, exhibiting a mean log2FC of − 0.52. Hence, whereas SB-699551 or docetaxel individually reduced xenograft growth during the treatment period the combination of both agents shrank the xenografts.

To determine the phenotypic consequences of treatment with SB-699551 or docetaxel alone or in combination, we stained FFPE xenograft sections from each cohort with hematoxylin and eosin (H&E) (Fig. 6e; Supplementary Figure 4). Tumor specimens collected from vehicle-treated mice recapitulated the histological features characteristic of HCC1954 xenografts and the majority of xenografts comprised a high tumor cell density [18]. Sections of tumors resected from docetaxel treated mice appeared histologically similar to those of the vehicle treated mice, albeit with a reduced tumor cell density. The xenografts of SB-699551 treated mice contained large, fibrotic areas that were devoid of tumor cells (red arrows) and displayed a decrease in tumor cell density. The xenografts of mice treated with docetaxel and SB-699551 also contained fibrotic regions and a reduced tumor cell density, but the “intact” regions of several xenografts comprised a considerable number of ghost cells and cells with shrunken, pyknotic nuclei (green arrows), features of cells that have undergone apoptosis.

To determine whether the characteristic histological phenotype after combination therapy was indeed due to increased tumor cell apoptosis, we stained the tumor sections using terminal deoxynucleotidyl transferase dUTP nick end labeling (TUNEL), which marks cells harboring nicked genomic DNA, a feature of cell death due to apoptosis, necrosis and autolytic cell death. Interestingly, whereas tumor xenografts isolated from mice treated with SB-699551 or docetaxel displayed only a subtle increase in the frequency of TUNEL-positive cells, their frequency was markedly increased in tumors isolated from mice treated with both compounds (Fig. 6f; Supplementary Figure 4). Taken together these results suggest that tumor shrinkage induced by the combination of SB-699551 and docetaxel may be due to an increase in tumor cell apoptosis.

## Discussion

The data presented herein strongly suggests that structurally unrelated selective 5-HT5A antagonists reduce the frequency of tumorsphere initiating cells by affecting the activity of 5-HT5A. The latter is supported by our pharmacological data demonstrating that the IC_50_ of the guanidine-type 5-HT5A antagonists in sphere forming assays directly correlated with their binding affinity for 5-HT5A [24, 36]. Moreover, CRISPR-Cas9 mediated mutagenesis of *HTR5A* reduced the frequency of tumorsphere initiating cells and that of BTIC using in vitro and ex vivo assays, thus mimicking the effect of SB-699551. Hence both pharmacological and genetic means of reducing 5-HT5A activity resulted in the same phenotypic consequences in breast tumor cell lines, suggesting that 5-HT5A is indeed required for BITC survival.

We also found that SB-699551 reduces the growth rate of human breast tumor xenografts in NOD/SCID mice when administered alone and shrinks the xenografts in combination with docetaxel. Histological examination and TUNEL assays revealed an increase in the frequency of apoptotic tumor cells in the xenografts of mice treated with a combination of both agents. The effect of each compound on tumor growth is consistent with SB-699551 and docetaxel targeting BTIC and their non-tumorigenic progeny respectively. Whereas these experiments were limited in terms of pharmacokinetic analysis of the circulating SB-699551 plasma concentration, previous studies have reported that administering a subcutaneous 3 mg/kg dose of SB-699551 in guinea pigs results in a C_max_ of approximately 663 nM [37]. These data suggest that the IC_50_ values from our in vitro assays are pharmacologically relevant in vivo in rodent models.

Our phosphoproteomic experiments established that exposure of breast tumor cells to SB-699551 curtails Gα_i/o_ signaling and that of the PI3K/AKT/mTOR pathway, lending further evidence of its on-target activity (Supplementary Figure 6). Previous studies have shown that 5-HT5A couples to Gα_i/o_ [23] and also signals via β-arrestin-2 [38] although the downstream effectors that mediate β-arrestin signaling have not been reported. Importantly, ligands of GPCRs, including 5-HTRs, can exhibit signaling bias toward effector proteins including the β-arrestins [39, 40]. We speculate that the latter may explain why ASP5736 did not affect tumorsphere formation, despite being a structural analogue of AS2030680, which was active in this assay [22]. ASP5763 may be a biased ligand that traps 5-HT5A in a conformation incapable of signaling via a pathway required for tumorsphere initiating cell viability.

We found that selective inhibitors of PI3K, AKT and mTOR all mimicked the effect of SB-699551 using in vitro functional assays suggesting that 5-HT5A may regulate the activity of these kinases. Whereas studies have reported that PI3K, AKT or mTOR inhibitors have antineoplastic effects, resistance to these inhibitors frequently develops due to feedback mechanisms between mTOR and AKT [41]. For example, treatment of breast tumor cells with rapamycin reduces the phosphorylation of the mTOR targets S6K and 4EBP1 while simultaneously increasing the phosphorylation of AKT and eIF4E. The latter resistance mechanism can be overcome by concurrent treatment with AKT and mTOR inhibitors, which durably inhibit the PI3K/AKT/mTOR pathway [42, 43]. Hence inhibition of the mTOR and AKT pathways by SB-699551 may afford a therapeutic benefit akin to that of the combination of mTOR and AKT inhibitors.

Sustained reduction in mTOR phosphorylation can promote activation of Atg13 and the formation of the Unc-51-like-kinase-1/2 (ULK1/2) complex [44]. The latter process initiates cellular autophagy, which permits tumor cells to resist the cytostatic effects of small molecule mTOR inhibitors. Whether such a mechanism could provide tumor cells with resistance to SB-699551, which inhibits both mTOR and AKT signaling pathways, remains unknown. If such a resistance mechanism does indeed exist, small molecule inhibitors of ULK1 might be used as described [44].

Our data are not the first to suggest that PI3K signaling may regulate BTIC activity. A recent study showed that inhibition of PI3K or AKT1 reduces the frequency of BTIC-enriched CD44^High^/CD24^Low^ breast tumor cells by initiating their apoptosis via FOXO3a and Bim [45]. Moreover, inhibition of 5-HT5A with SB-699551 affects the survival of mouse embryonic hematopoietic progenitor cells by reducing the phosphorylation of AKT and FOXO1, and increasing the abundance of FOXO1 and Bim [35]. Hence our hypothesis that SB-699551 targets BTIC in part by inhibiting AKT/FOXO signaling is consistent with the reports of others.

Whereas the role of 5-HT5A in cancer is largely unknown, a recent study demonstrated that *HTR5A* was among the most differentially upregulated genes after hypoxia-mediated neuroendocrine differentiation of prostate tumor cells [46]. Neuroendocrine differentiation of prostate tumor cells is a process that selects for a highly aggressive tumor cell phenotype and increases the frequency of TIC [47], which may suggest that 5-HT5A activity in part regulates prostate TIC activity.

There are several 5-HTRs whose expression and/or activity have been implicated in TIC activity. A recent study utilized machine-learning to extract features of stemness from human pluripotent stem cells and their differentiated progeny in order to delineate pathways associated with tumor cell anaplasia [48]. Connectivity mapping that was used to identify compounds that target the established stemness indices identified 5-HT1A, 5-HT2A and 5-HT2C among several other molecular targets. Interestingly, ectopic expression of cDNAs encoding each of the latter 5-HTRs induces the transformation of mouse fibroblasts suggesting that 5-HTRs may act as proto-oncogenes [49–51]. Other studies have demonstrated that expression of 5-HTRs confers drug resistance to tumor cells, which is a commonly reported property of TIC [3, 5, 52]. For example, ectopic expression of HTR2C in melanoma cell lines confers resistance to MEK inhibitors [53], whereas expression of HTR1D in lung cancer cells provides them with resistance to EML-ALK inhibitors [54]. These data suggest that 5-HTR activity may be associated with several characteristics of TIC residing in different tumor types.

## Conclusions

In summary our data establish that structurally unrelated selective antagonists of 5-HT5A reduce BTIC frequency, an effect that is mimicked by genetic loss of the receptor. Selective inhibition of 5-HT5A activity with SB-699551 in NOD/SCID mice harboring breast tumor xenografts reduced their growth rate and acted in concert with cytotoxic chemotherapy to shrink the xenografts. Mechanistically SB-699551 reduced 5-HT5A activity by compromising its capacity to signal to downstream effectors known to be dysregulated in breast and other cancers. Collectively our data suggest that 5-HT5A is a suitable molecular target for anticancer drug development.

## Supplementary information

**Additional file 1: Figure S1.** Enantiomer selectivity of guanidine-type 5-HT5A antagonists measured by the PrestoBlue reduction cell viability assay. (A) IC_50_ curves of enantiopure guanidine-type 5-HT5A antagonists. Each point indicates the mean residual activity of tumor cells at each concentration of each inhibitor +/− the SEM. The blue curves show the effect of the (S) isomers whereas the red curves show the effect of the (R) isomers. (B) Bar graphs showing that the viability of the tumor cells was unaffected by treatment with G1A-(R) and G1B-(R) (red) at the approximate IC_50_ concentrations (2.7 μM) of their (S)-enantiomers (blue). Statistical significance was determined by one-way ANOVA and post-hoc Tukey’s tests [**** *p* < 0.0001; *** *p* = 0.005; ** *p* = 0.001; ns *p* > 0.05].

**Additional file 2: Figure S2**. Uncropped X-ray film from PPA. (A-B) Results of the PPA experiment after a short (A) or long (B) exposure to X-ray film. The phosphoprotein corresponding to each coordinate is listed in Supplementary Table 1.

**Additional file 3: Table S1**. Legend key for PPA.

**Additional file 4: Figure S3**. SB-699551 targets BTIC-enriched MMTV-*Neu*-derived tumorspheres with greater potency than biologically matched normal mammospheres. (A-B) IC_50_ curves of sphere forming assays (A) and PrestoBlue cell viability assays (B) demonstrating that several concentrations of SB-699551 preferentially target tumorsphere-derived cells (TMS; red) by comparison to mammospheres (MMS; blue). (C-D) Approximate IC_50_ (500 nM) of SB-699551 in TMS has no effect on MMS-formation (C) or MMS PrestoBlue reduction (D). Error bars represent the SEM. Statistical significance was determined using an unpaired t-test [*** *p* = 0.0005; ** *p* = 0.0082; ns p > 0.05].

**Additional file 5: Table S2**. Maximum tolerated dose of SB-699551 in combination with docetaxel (10 mg/kg) in each of two mice.

**Additional file 6: Figure S4**. The combination of SB-699551 and docetaxel increases the frequency of apoptotic tumor cells in vivo. (A) Histological sections of 3 tumors from each cohort were prepared and stained with H&E. The red arrows indicate fibrotic areas that are devoid of tumor cells. The green arrows identify cells undergoing apoptosis with a characteristic shrunken appearance and pyknotic nuclei. The top 3 rows were imaged at 200X magnification, whereas the bottom row was imaged at 400X. (B) Tumor sections were stained using the TUNEL assay and imaged under the 4′,6-diamidino-2-phenylindole (DAPI) channel (top row), the fluorescein isothiocyanate (FITC) channel (middle row) and merged (bottom row). Images were taken at 100X magnification. Scale bars represents 100 μm.

**Additional file 7: Figure S5**. Mean bodyweight of mice before and after two weeks of treatment in vivo. SB-699551, docetaxel or their combination resulted in minimal weight loss (< 1 g on average). Mouse activity level remained normal throughout the experiment. Error bars represent the SEM.

**Additional file 8: Figure S6**. Schematic of a proposed mechanism whereby SB-699551 affects signalling downstream of 5-HT5A.

**Additional file 9: Figure S7**. In silico analysis of Cas9-mediated INDELs in *HTR5A.* (A) The frequency of in-frame (mutations) and frameshift mutations among INDEL-containing reads in the genomic DNA of dox-induced clones. (B) The upper and lower limits of amino acid insertions/deletions in mutant reads. (C) Examples of INDEL translational consequences. Red arrows indicate corresponding Cas9 cleavage site in genomic DNA. The red X indicates a deleted amino acid whereas amino acids insertions are coloured blue. The * indicates a nonsense mutation.

**Additional file 10: Figure S8**. Uncropped western blots used in Fig. 3. Blots were imaged using the LI-COR Biosciences Odyssey Platform. Each blot was imaged under the 700 nM channel (left), which displays the molecular weight markers and the protein of interest, and under the 800 nM channel (right), which displays the α-Tubulin loading control. Blots were cropped where indicated by the horizontal red lines.

**Additional file 11.** Supplementary Methods.

## Data Availability

Not applicable.

## Abbreviations

AC: Adenylyl cyclase
ANOVA: Analysis of variance
ATF1: Activating transcription factor 1
BTIC: Breast tumor initiating cell
cAMP: Cyclic adenosine monophosphate
CRISPR: Clustered interspersed palindromic repeats
CREB: cAMP-response element binding protein
EMT: Epithelial-to-mesenchymal transition
ER: Estrogen receptor
FFPE: Formalin fixed paraffin embedded
FOXO: Forkhead box protein
GPCR: G-protein coupled receptor
HER2: Human epidermal growth factor receptor 2
H&E: Hematoxylin and eosin
HTR5A: 5-hydroxytryptamine receptor 5A gene
iCas9: Inducible cas9
IC_50_: Half-maximal inhibitory concentration
iKO: Inducible knockout
INDELs: Insertions/deletions
MAO-A: Monoamine oxidase A
mg/kg: milligrams per kilogram body weight
MOI: Multiplicity of infection
mTOR: Mammalian target of rapamycin
nM: nanomolar
NOD/SCID: Non-obese diabetic/severe combined immune deficient
NT: Non-targeting
PRAS40: Proline-rich Akt substrate
PPA: Proteome profiler array
PI3K: Phosphoinositide 3-kinase
SERT: Serotonin reuptake transporter
SEM: Standard error of the mean
TIC: Tumor initiating cell
TPH1: Tryptophan hydroxylase 1
TUNEL: Terminal deoxynucleotidyl transferase dUTP nick end labeling
WNK1: with no lysine kinase 1
5-HT: 5-hydroxytryptamine
5-HT5A: 5-hydroxytryptamine receptor 5A
5-HTRs: 5-hydroxytryptamine receptors
