# Supplementary figures and images for: Antagonists of the serotonin receptor 5A target human breast tumor initiating cells
Source: BMC Cancer. 2020 Aug 5;20:724. doi: 10.1186/s12885-020-07193-6 (PMC7404930; doi:10.1186/s12885-020-07193-6)

**A**

**MCF-7**

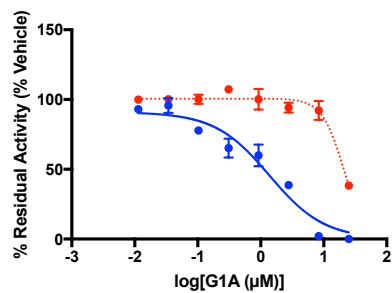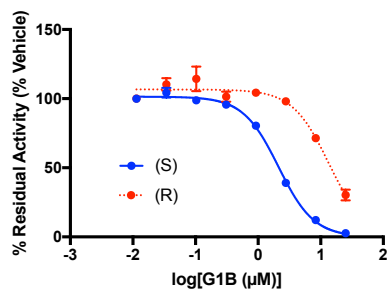

**B**

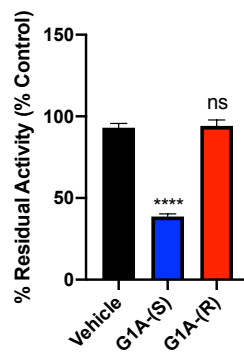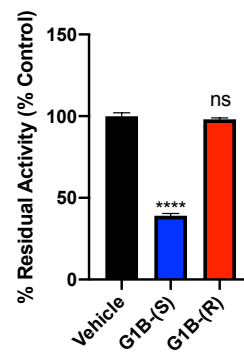

**HCC1954**

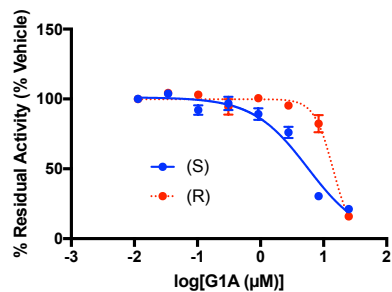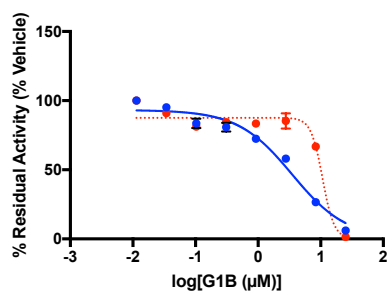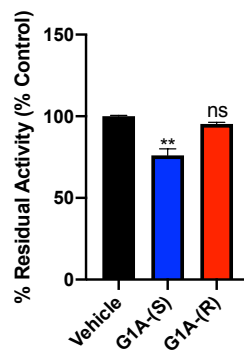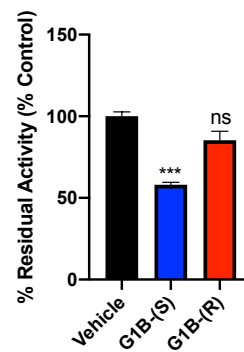

Supplement: Supplementary file 1 — Additional file 1: Figure S1. Enantiomer selectivity of guanidine-type 5-HT5A antagonists measured by the PrestoBlue reduction cell viability assay. (A) IC50 curves of enantiopure guanidine-type 5-HT5A antagonists. Each point indicates the mean residual activity of tumor cells at each concentration of each inhibitor +/− the SEM. The blue curves show the effect of the (S) isomers whereas the red curves show the effect of the (R) isomers. (B) Bar graphs showing that the viability of the tumor cells was unaffected by treatment with G1A-(R) and G1B-(R) (red) at the approximate IC50 concentrations (2.7 μM) of their (S)-enantiomers (blue). Statistical significance was determined by one-way ANOVA and post-hoc Tukey’s tests [**** p < 0.0001; *** p = 0.005; ** p = 0.001; ns p > 0.05]. [file 12885_2020_7193_MOESM1_ESM.pdf]

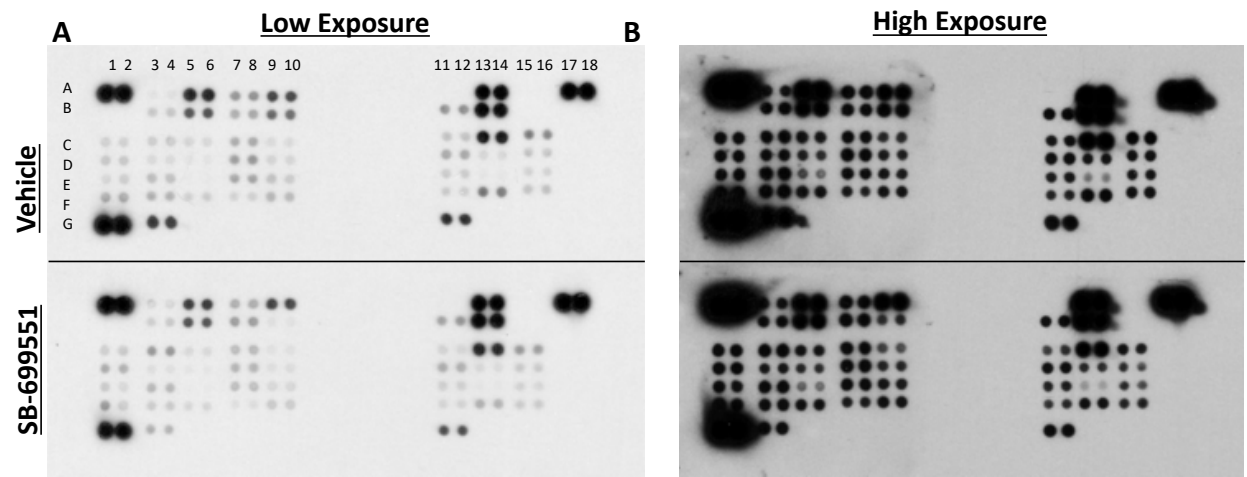

Supplement: Supplementary file 2 — Additional file 2: Figure S2. Uncropped X-ray film from PPA. (A-B) Results of the PPA experiment after a short (A) or long (B) exposure to X-ray film. The phosphoprotein corresponding to each coordinate is listed in Supplementary Table 1. [file 12885_2020_7193_MOESM2_ESM.pdf]

## Sphere Formation

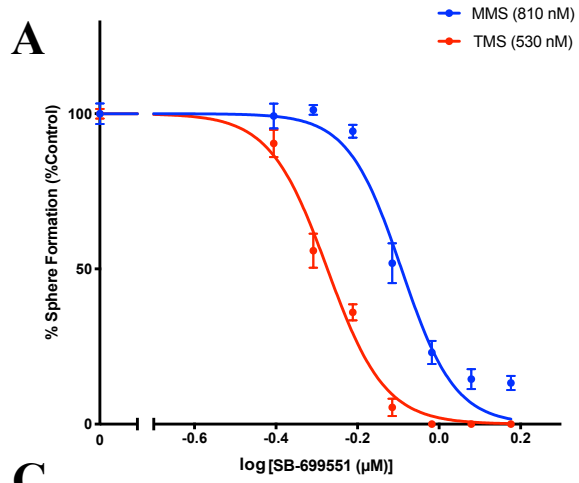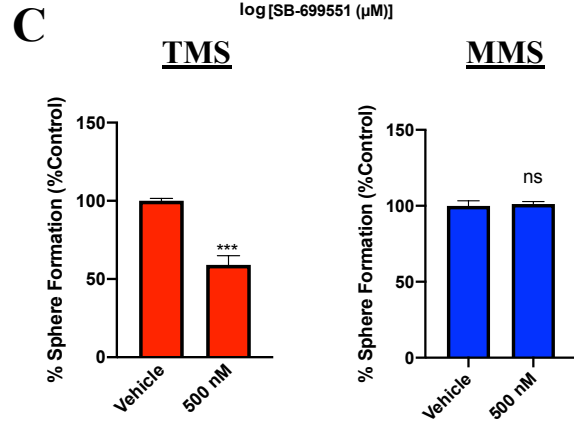

## PrestoBlue Reduction

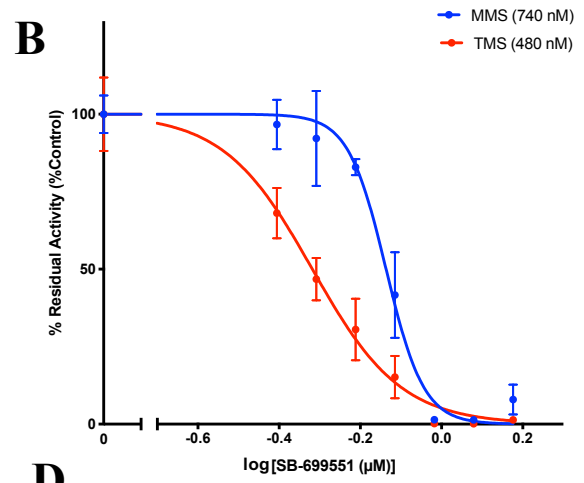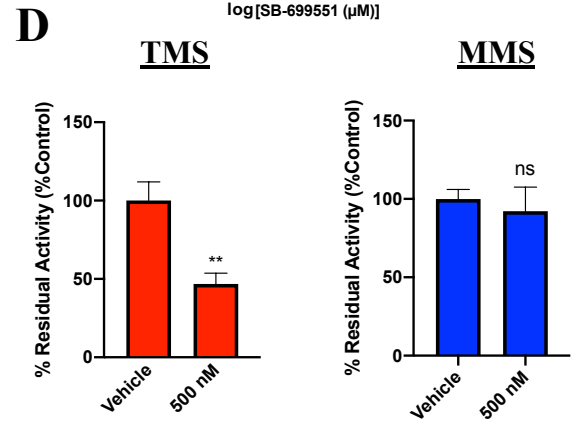

Supplement: Supplementary file 4 — Additional file 4: Figure S3. SB-699551 targets BTIC-enriched MMTV-Neu-derived tumorspheres with greater potency than biologically matched normal mammospheres. (A-B) IC50 curves of sphere forming assays (A) and PrestoBlue cell viability assays (B) demonstrating that several concentrations of SB-699551 preferentially target tumorsphere-derived cells (TMS; red) by comparison to mammospheres (MMS; blue). (C-D) Approximate IC50 (500 nM) of SB-699551 in TMS has no effect on MMS-formation (C) or MMS PrestoBlue reduction (D). Error bars represent the SEM. Statistical significance was determined using an unpaired t-test [*** p = 0.0005; ** p = 0.0082; ns p > 0.05]. [file 12885_2020_7193_MOESM4_ESM.pdf]

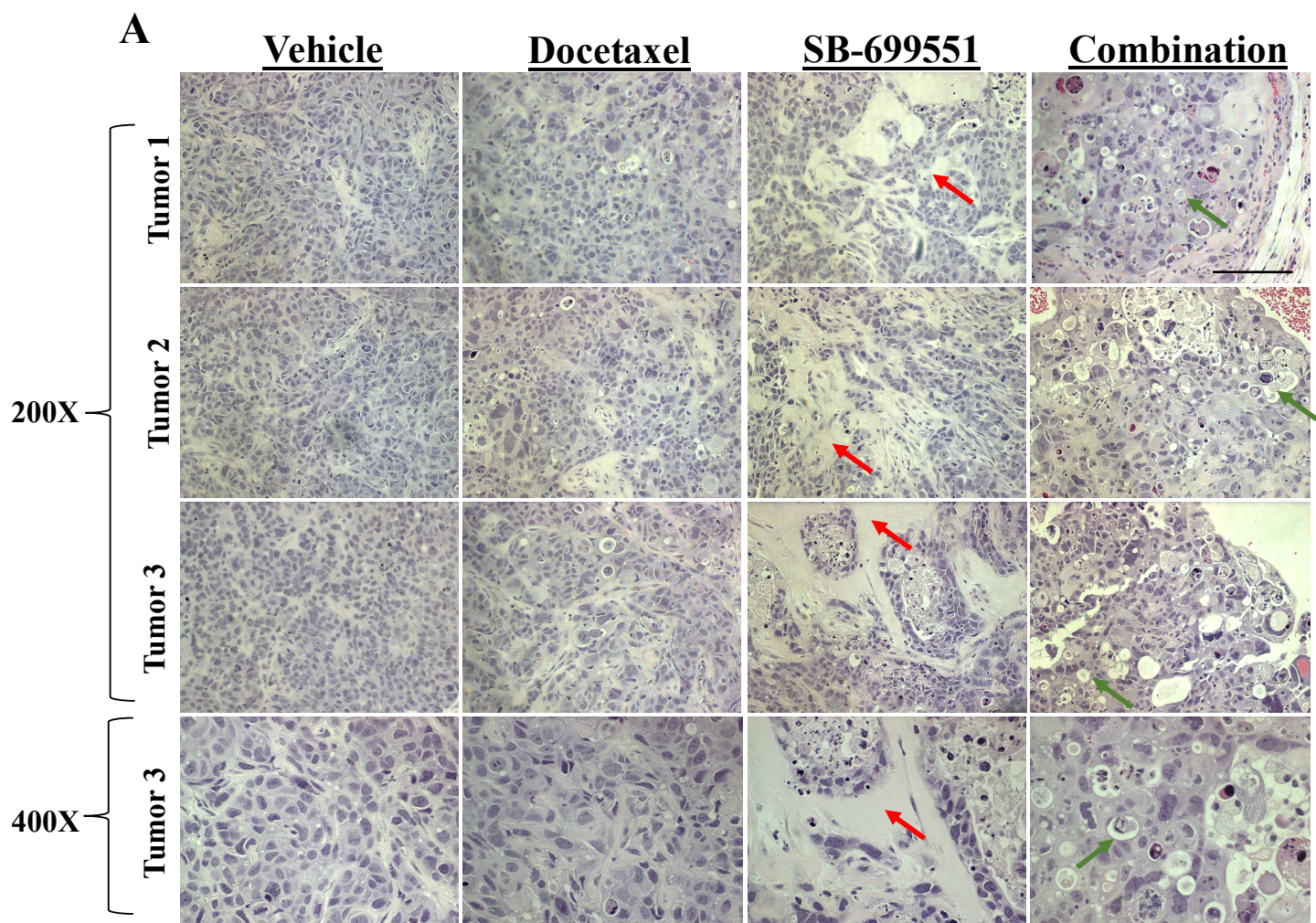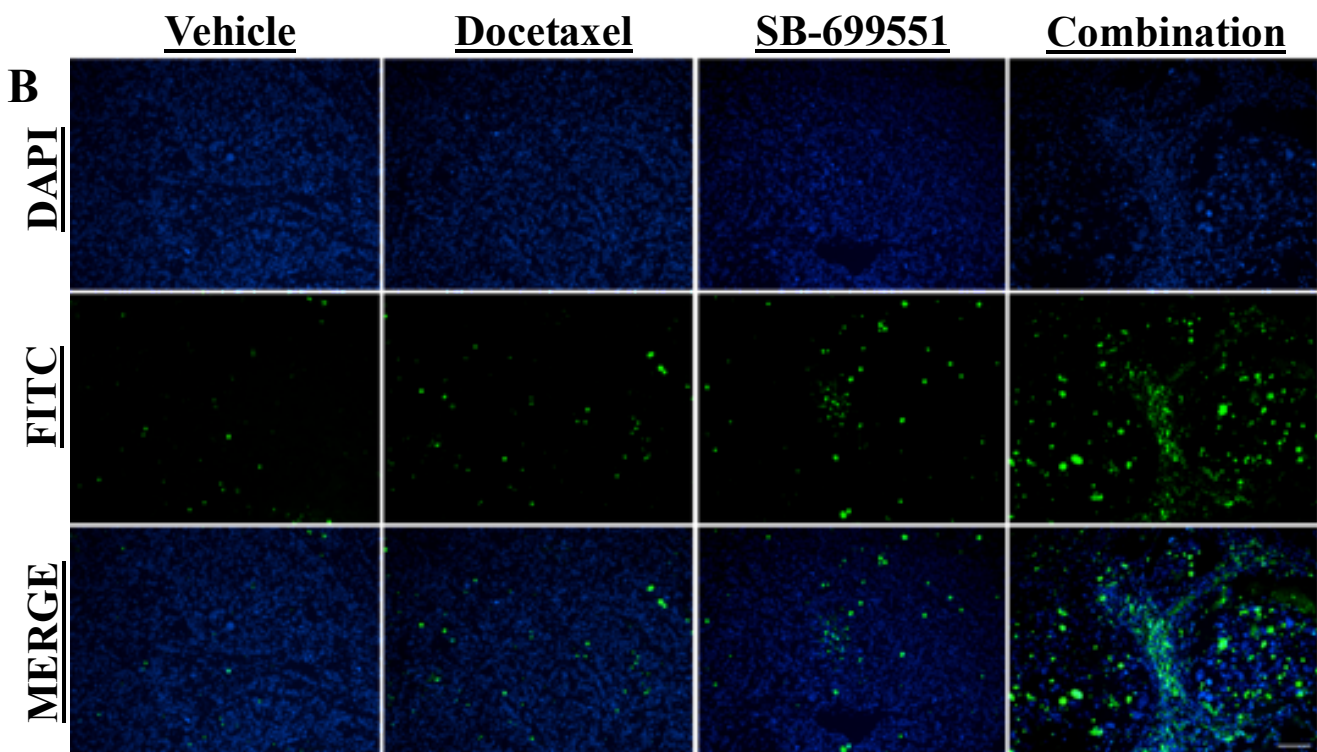

Supplement: Supplementary file 6 — Additional file 6: Figure S4. The combination of SB-699551 and docetaxel increases the frequency of apoptotic tumor cells in vivo. (A) Histological sections of 3 tumors from each cohort were prepared and stained with H&E. The red arrows indicate fibrotic areas that are devoid of tumor cells. The green arrows identify cells undergoing apoptosis with a characteristic shrunken appearance and pyknotic nuclei. The top 3 rows were imaged at 200X magnification, whereas the bottom row was imaged at 400X. (B) Tumor sections were stained using the TUNEL assay and imaged under the 4′,6-diamidino-2-phenylindole (DAPI) channel (top row), the fluorescein isothiocyanate (FITC) channel (middle row) and merged (bottom row). Images were taken at 100X magnification. Scale bars represents 100 μm. [file 12885_2020_7193_MOESM6_ESM.pdf]

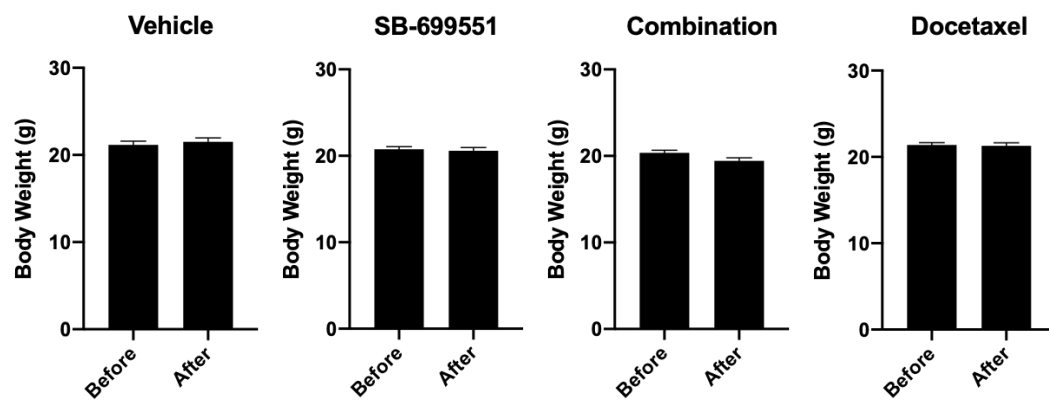

Supplement: Supplementary file 7 — Additional file 7: Figure S5. Mean bodyweight of mice before and after two weeks of treatment in vivo. SB-699551, docetaxel or their combination resulted in minimal weight loss (< 1 g on average). Mouse activity level remained normal throughout the experiment. Error bars represent the SEM. [file 12885_2020_7193_MOESM7_ESM.pdf]

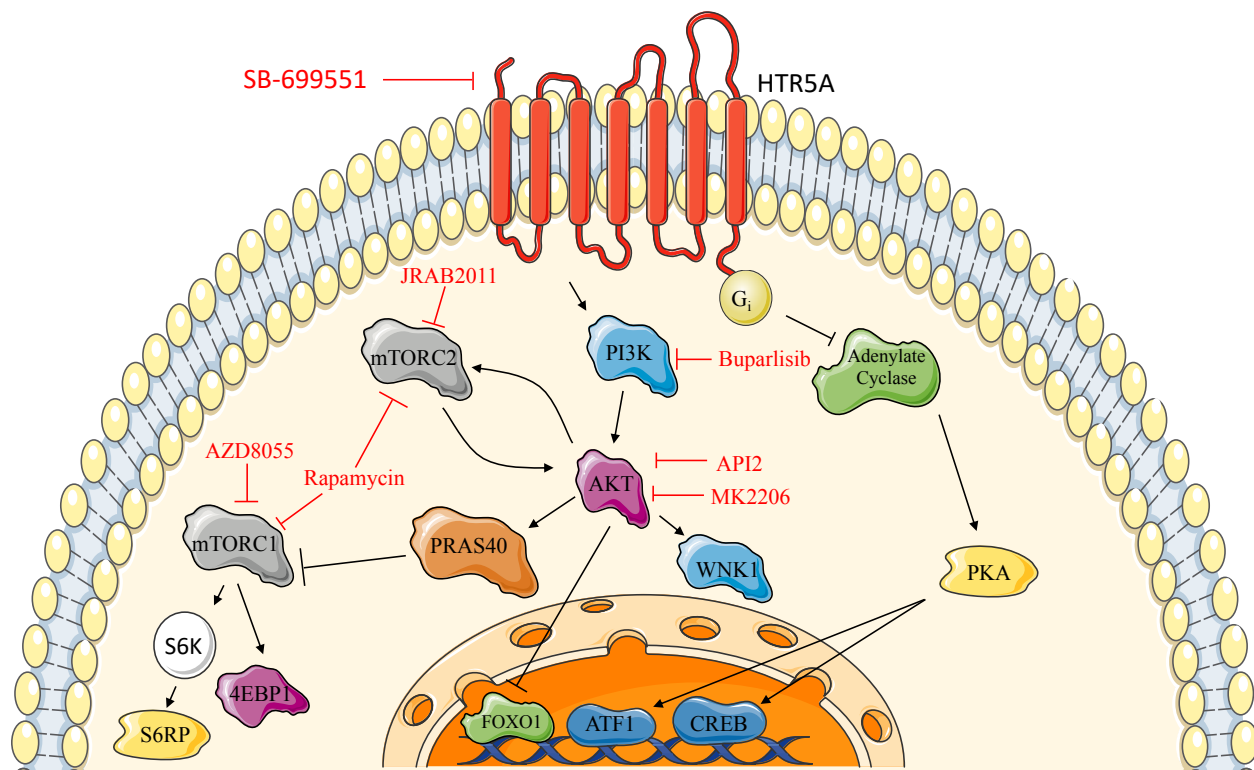

Supplement: Supplementary file 8 — Additional file 8: Figure S6. Schematic of a proposed mechanism whereby SB-699551 affects signalling downstream of 5-HT5A. [file 12885_2020_7193_MOESM8_ESM.pdf]
