# Supplementary material for: Antagonists of the serotonin receptor 5A target human breast tumor initiating cells
Source: BMC Cancer. 2020 Aug 5;20:724. doi: 10.1186/s12885-020-07193-6 (PMC7404930; doi:10.1186/s12885-020-07193-6)
Supplement: Supplementary file 3 — Additional file 3: Table S1. Legend key for PPA. [file 12885_2020_7193_MOESM3_ESM.pdf]

Supplementary Table 1. Legend key for PPA.

Coordinate	Target	Phosphorylation	Coordinate	Target	Phosphorylation
**A-A1, A2**	Reference	-	**A-D9, D10**	STAT5a	Y694
**A-A3, A4**	p38α	T180/Y182	**B-D11, D12**	p70 S6 Kinase	T421/S424
**A-A5, A6**	ERK1/2	T202/Y204, T185/ Y187	**B-D13, D14**	RSK1/2/3	S380/S386/S377
**A-A7, A8**	JNK 1/2/3	T183/Y185, T221/ Y223	**B-D15, D16**	eNOS	S1177
**A-A9, A10**	GSK-3α/β	S21/S9	**A-E1, E2**	Fyn	Y420
**B-A13, A14**	p53	S392	**A-E3, E4**	Yes	Y426
**B-A17, A18**	Reference	-	**A-E5, E6**	Fgr	Y412
**A-B3, B4**	EGF R	Y1086	**A-E7, E8**	STAT6	Y641
**A-B5, B6**	MSK1/2	S376/S360	**A-E9, E10**	STAT5b	Y699
**A-B7, B8**	AMPKα1	T183	**B-E11, E12**	STAT3	Y705
**A-B9, B10**	Akt 1/2/3	S473	**B-E13, E14**	p27	T198
**B-B11, B12**	Akt 1/2/3	T308	**B-E15, E16**	PLC-γ1	Y783
**B-B13, B14**	p53	S46	**A-F1, F2**	Hck	Y411
**A-C1, C2**	TOR	S2448	**A-F3, F4**	Chk-2	T68
**A-C3, C4**	CREB	S133	**A-F5, F6**	FAK	Y397
**A-C5, C6**	HSP27	S78/S82	**A-F7, F8**	PDGF Rβ	Y751
**A-C7, C8**	AMPKα2	T172	**A-F9, F10**	STAT5a/b	Y694/Y699
**A-C9, C10**	β-Catenin	-	**B-F11, F12**	STAT3	S727
**B-C11, C12**	p70 S6 Kinase	T389	**B-F13, F14**	WNK1	T60
**B-C13, C14**	p53	S15	**B-F15, F16**	PYK2	Y402
**B-C15, C16**	c-Jun	S63	**A-G1, G2**	Reference	-
**A-D1, D2**	Src	Y419	**A-G3, G4**	PRAS40	T246
**A-D3, D4**	Lyn	Y397	**A-G9, G10**	PBS (Negative Control)	-
**A-D5, D6**	Lck	Y394	**B-G11, G12**	HSP60	-
**A-D7, D8**	STAT2	Y689	**B-G17, G18**	PBS (Negative Control)	-

Information in this table was accessed via the user manual for ARY003B (R&D Systems) via the weblink: https://www.rndsystems.com/products/proteome-profiler-human-phospho-kinase-array-kit_ary003b
