# Supplementary material for: Antagonists of the serotonin receptor 5A target human breast tumor initiating cells
Source: BMC Cancer. 2020 Aug 5;20:724. doi: 10.1186/s12885-020-07193-6 (PMC7404930; doi:10.1186/s12885-020-07193-6)
Supplement: Supplementary file 5 — Additional file 5: Table S2. Maximum tolerated dose of SB-699551 in combination with docetaxel (10 mg/kg) in each of two mice. [file 12885_2020_7193_MOESM5_ESM.pdf]

Supplementary Table 2. Maximum tolerated dose of SB-699551 in combination with docetaxel (10 mg/kg) in each of two mice.

SB-699551 Dose (i.p.)	Body Mass at Endpoint (g)	Observations
Vehicle - 1<br>Vehicle - 2	21.9<br>21.7	Groomed fur, phenotype normal, active
12.5 mg/kg - 1<br>12.5 mg/kg - 2	19.7<br>19.5	Groomed fur, phenotype normal, active
25 mg/kg - 1<br>25 mg/kg - 2	18.5<br>18.6	Slight fur ruffling, phenotype normal, active
37.5 mg/kg - 1<br>37.5 mg/kg - 2	16.6<br>19.5	Slight fur ruffling decreased body condition, extremely active. Condition deteriorated initially in 37.5 mg/kg - 1 due to diarrhea but the mouse recovered.
50 mg/kg - 1<br>50 mg/kg - 2	14.6<br>17.4	Mice steadily lost body condition throughout treatment. Low activity.

NOD/SCID mice weighing 18 g (+/- 1g) were treated for two weeks consecutively according to the treatment schedule outlined in figure 6A. Mice were observed daily for their phenotype, activity level, body composition, alertness and hydration level. Bodyweight of mice at endpoint was determined using a digital scale (sensitivity 0.1g).
