# Supplementary material for: Antagonists of the serotonin receptor 5A target human breast tumor initiating cells
Source: BMC Cancer. 2020 Aug 5;20:724. doi: 10.1186/s12885-020-07193-6 (PMC7404930; doi:10.1186/s12885-020-07193-6)
Supplement: Supplementary file 9 — Additional file 9: Figure S7. In silico analysis of Cas9-mediated INDELs in HTR5A. (A) The frequency of in-frame (mutations) and frameshift mutations among INDEL-containing reads in the genomic DNA of dox-induced clones. (B) The upper and lower limits of amino acid insertions/deletions in mutant reads. (C) Examples of INDEL translational consequences. Red arrows indicate corresponding Cas9 cleavage site in genomic DNA. The red X indicates a deleted amino acid whereas amino acids insertions are coloured blue. The * indicates a nonsense mutation. [file 12885_2020_7193_MOESM9_ESM.pdf]

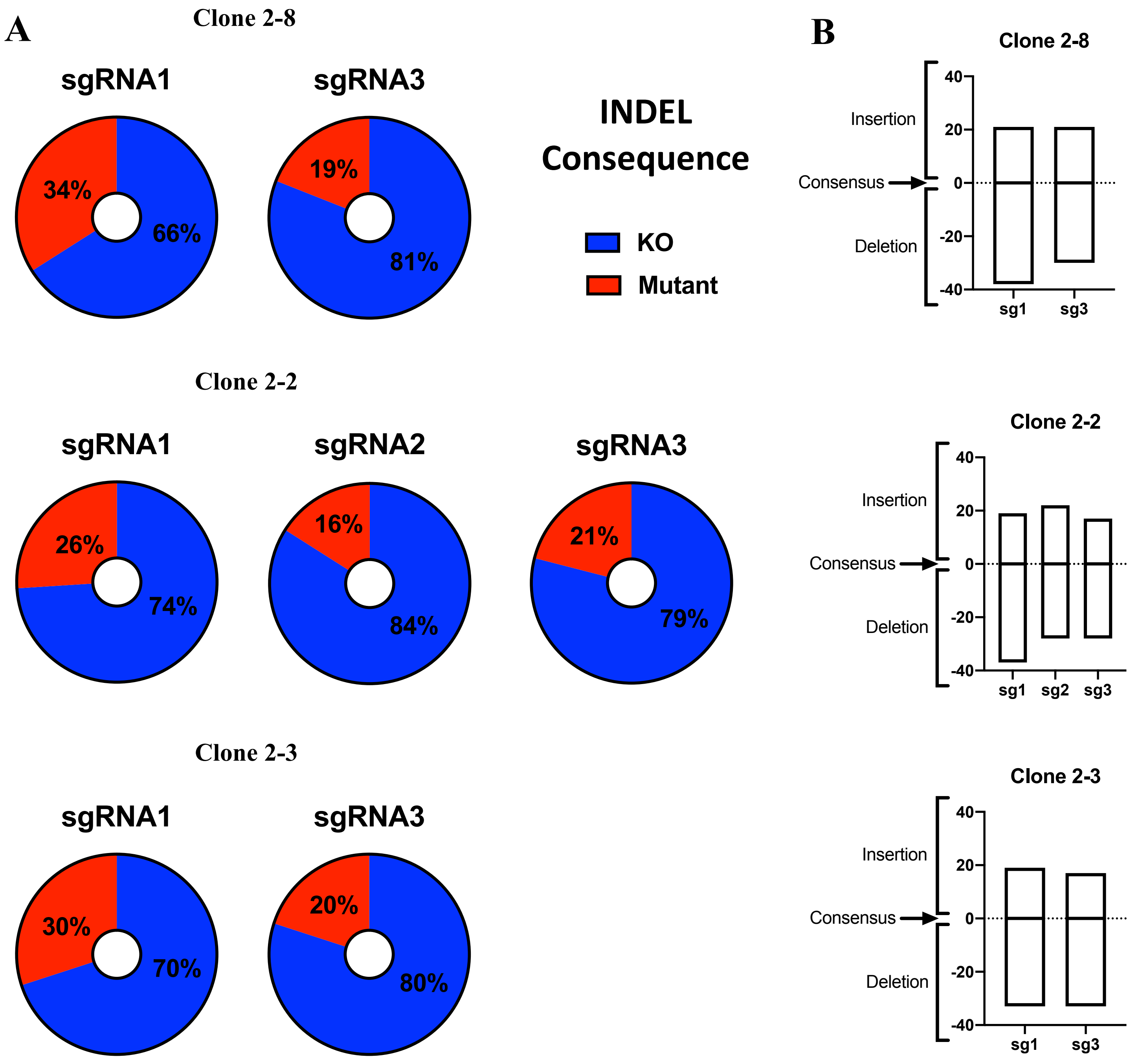


**C**

VFGVLILTLLGFLVAATFAWNLLVLATILRVRTFHRVPHNLVASMAVSDVLVAALVMPLSLV **sgRNA1 Amplicon Consensus**

**IN FRAME MUTANTS:**

(i) VFGVLILTLLGFLVAATFAWNLLVLATILRVXXXXXXPHNLVASMAVSDVLVAALVMPLSLV
(ii) VFGVLILTLLGFLVAATFAWNLXXXXXXXXXXXXXXXXXXXXXXXXXXXXLVAALVMPLSLV
(iii) VFGVLILSLLGFLVAATFAWNLLVLATILXSQQSTTTPMRMPTMRAARCSFCVRTFHRVPHNLVASTAVSDALVAALVMPLSLV

**FRAMESHIFT MUTANTS**

(iv) VFGVLILTLLGFLVAATFAWNLLVLATILPRWSCR*AW
(v) VFGVLILTLLGFLVAATFAWNLLVLATIRRAGHAAEPG
