# Supplementary material for: Antagonists of the serotonin receptor 5A target human breast tumor initiating cells
Source: BMC Cancer. 2020 Aug 5;20:724. doi: 10.1186/s12885-020-07193-6 (PMC7404930; doi:10.1186/s12885-020-07193-6)

MCF-7 Figure 3 D-E

Blot 1

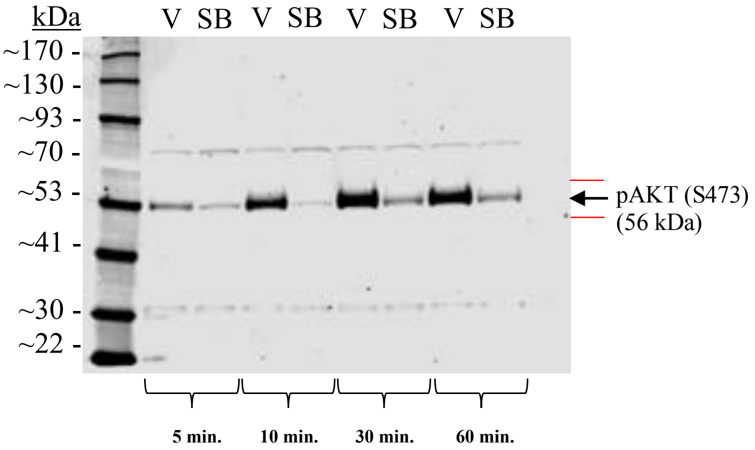

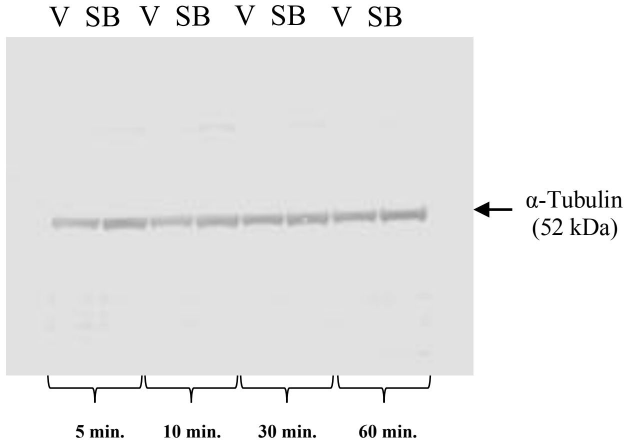

Blot 2

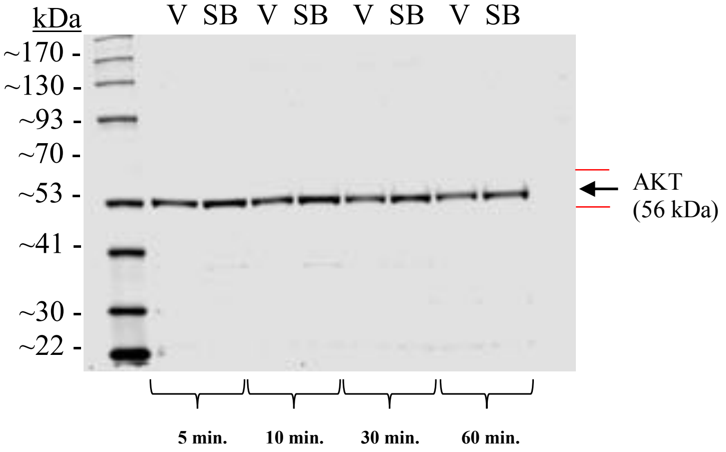

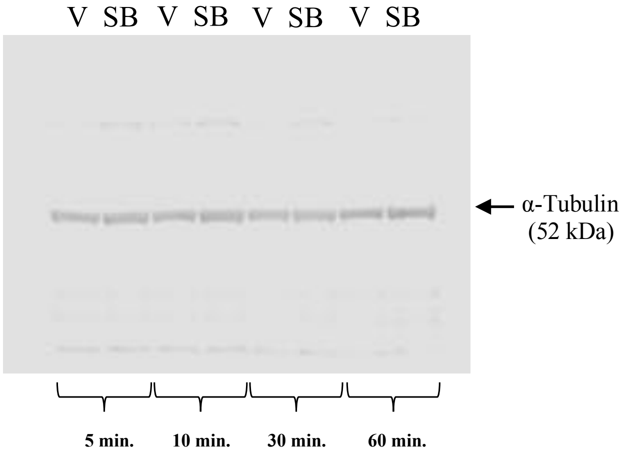

Blot 3

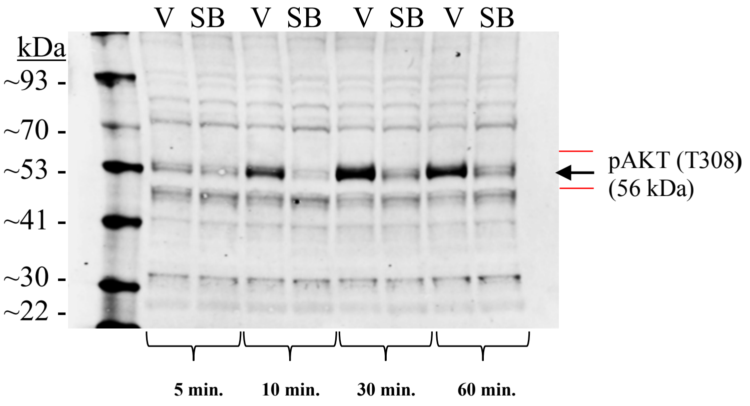

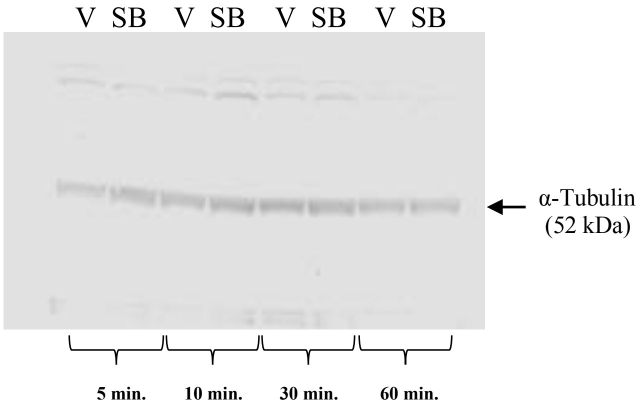

Blot 4

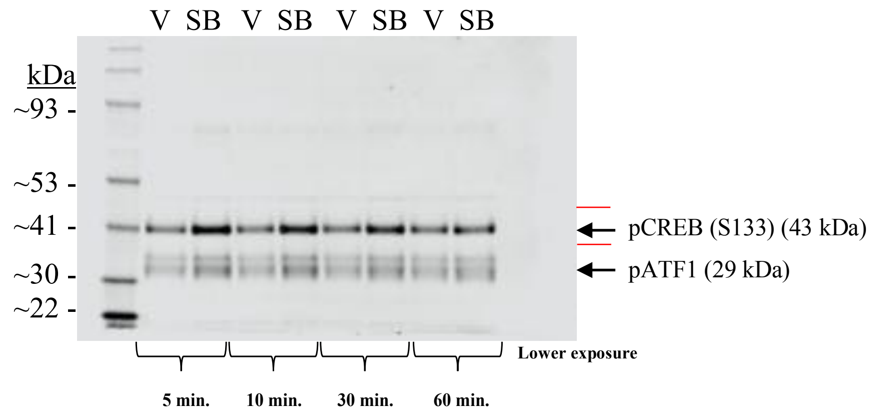
V SB V SB V SB V SB
kDa
~93 -
~53 -
~41 -
~30 -
~22 -
pCREB (S133) (43 kDa)
pATF1 (29 kDa)
Lower exposure
5 min.
10 min.
30 min.
60 min.

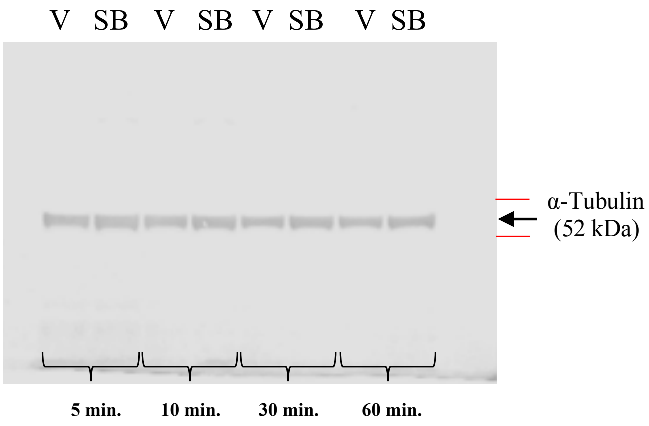
V SB V SB V SB V SB
α-Tubulin
(52 kDa)
5 min.
10 min.
30 min.
60 min.

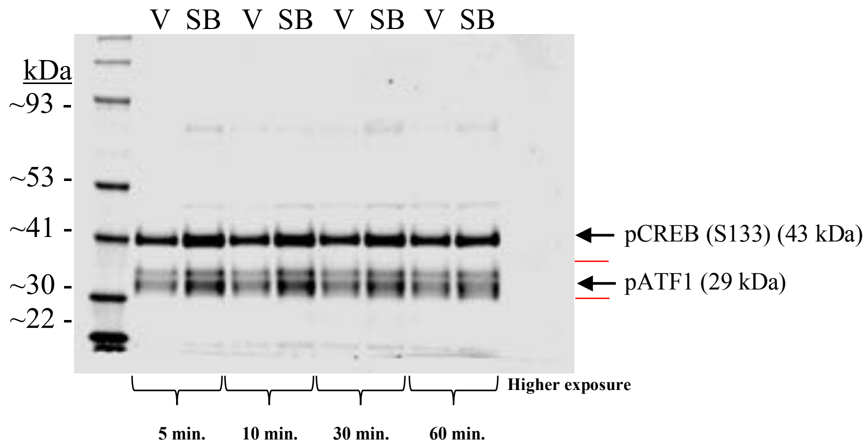
V SB V SB V SB V SB
kDa
~93 -
~53 -
~41 -
~30 -
~22 -
pCREB (S133) (43 kDa)
pATF1 (29 kDa)
Higher exposure
5 min.
10 min.
30 min.
60 min.

Blot 5

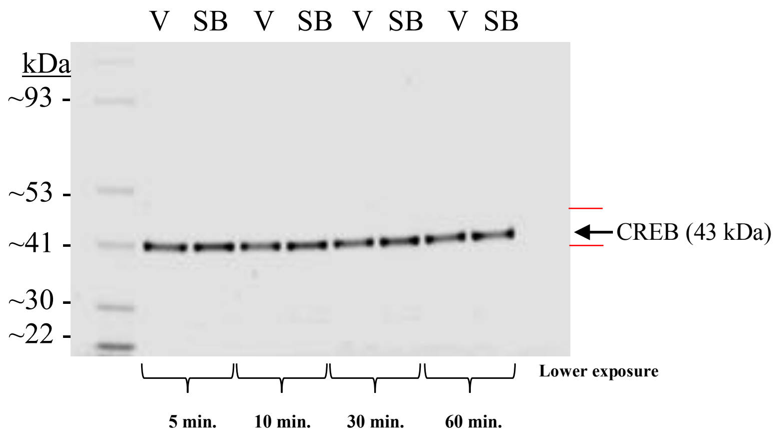
V SB V SB V SB V SB
kDa
~93 -
~53 -
~41 -
~30 -
~22 -
CREB (43 kDa)
Lower exposure
5 min.
10 min.
30 min.
60 min.

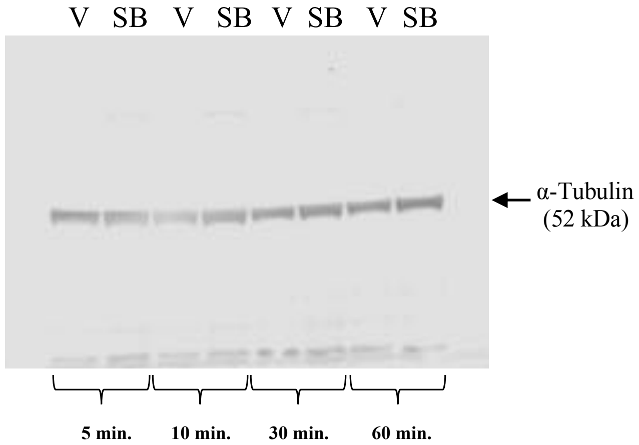
V SB V SB V SB V SB
α-Tubulin
(52 kDa)
5 min.
10 min.
30 min.
60 min.

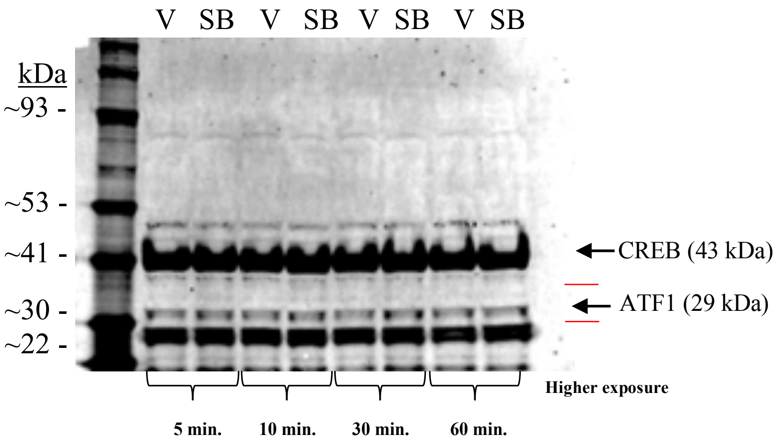
V SB V SB V SB V SB
kDa
~93 -
~53 -
~41 -
~30 -
~22 -
CREB (43 kDa)
ATF1 (29 kDa)
Higher exposure
5 min.
10 min.
30 min.
60 min.

Blot 6

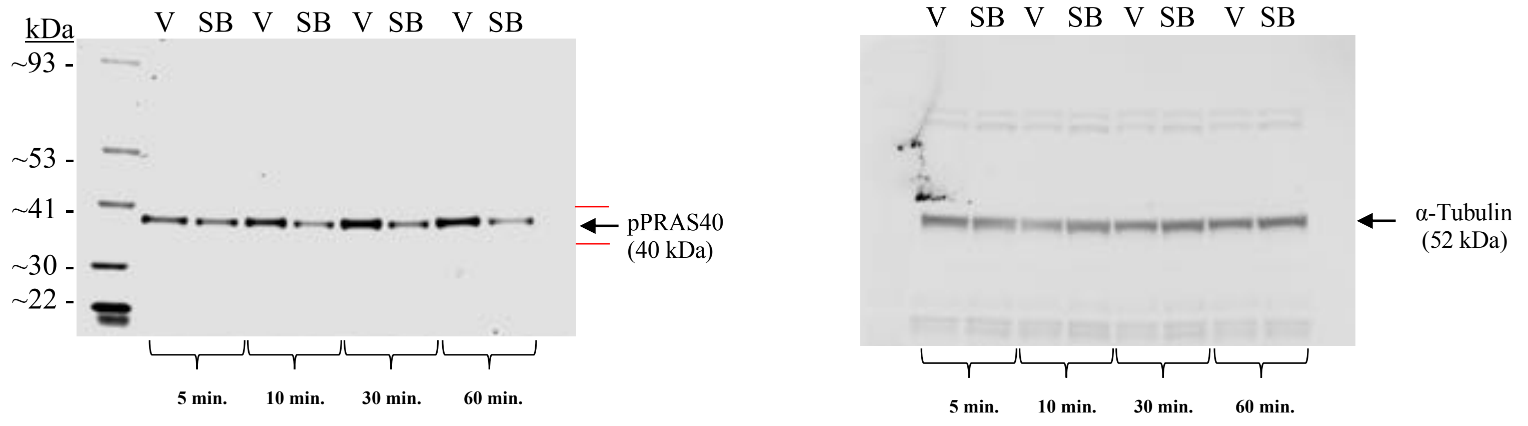

Blot 7

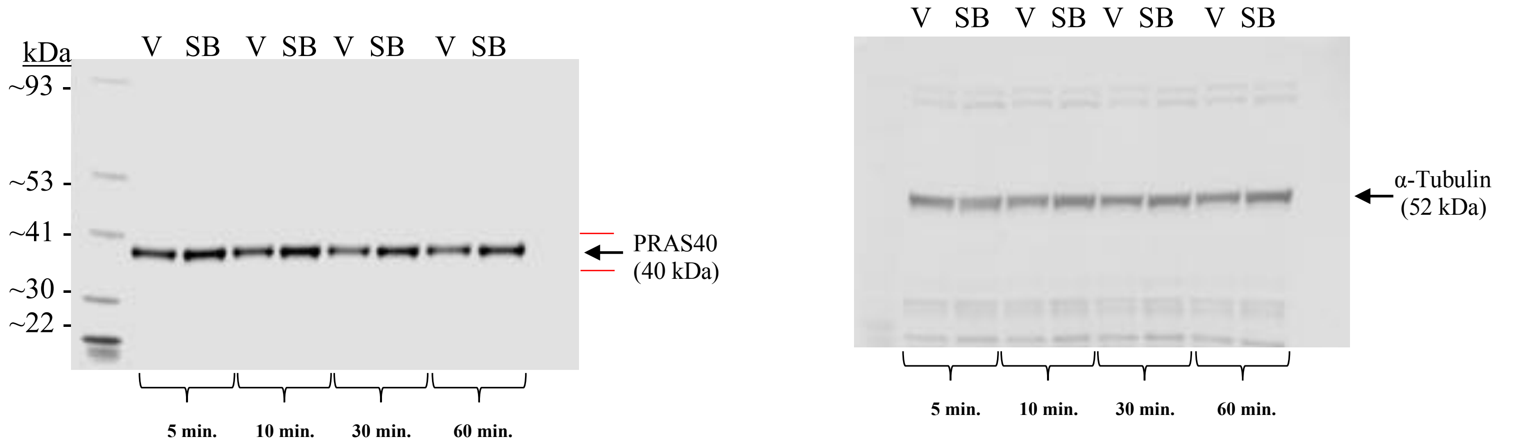

Blot 8

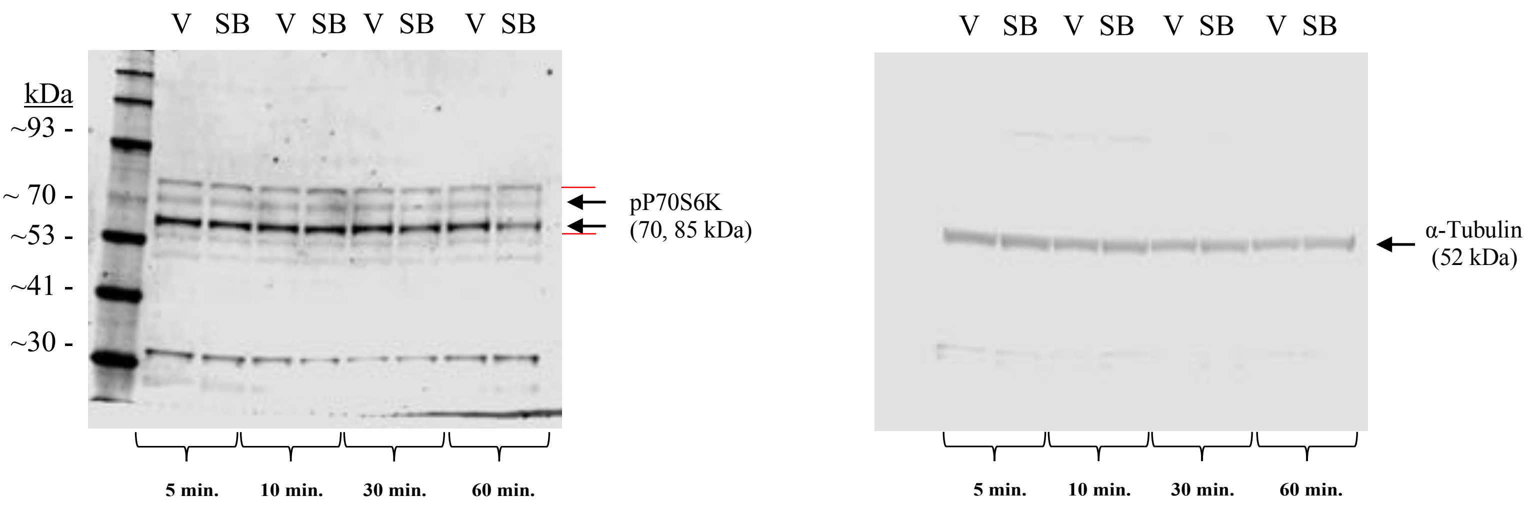

Blot 9

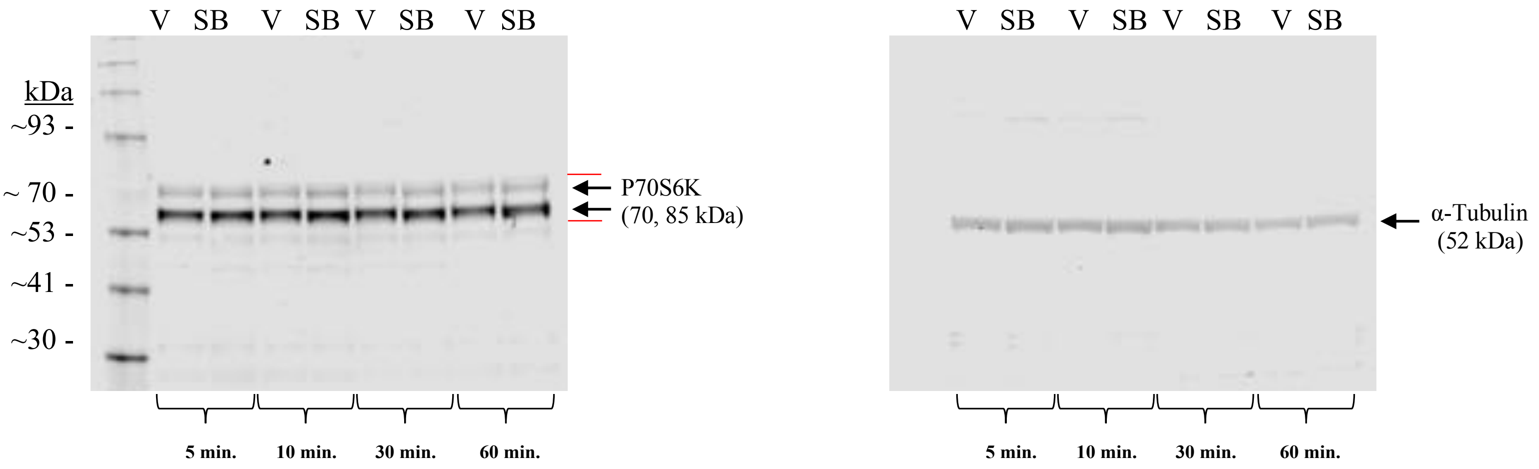

MDA-MB-157 Figure 3D-E

Blot 10

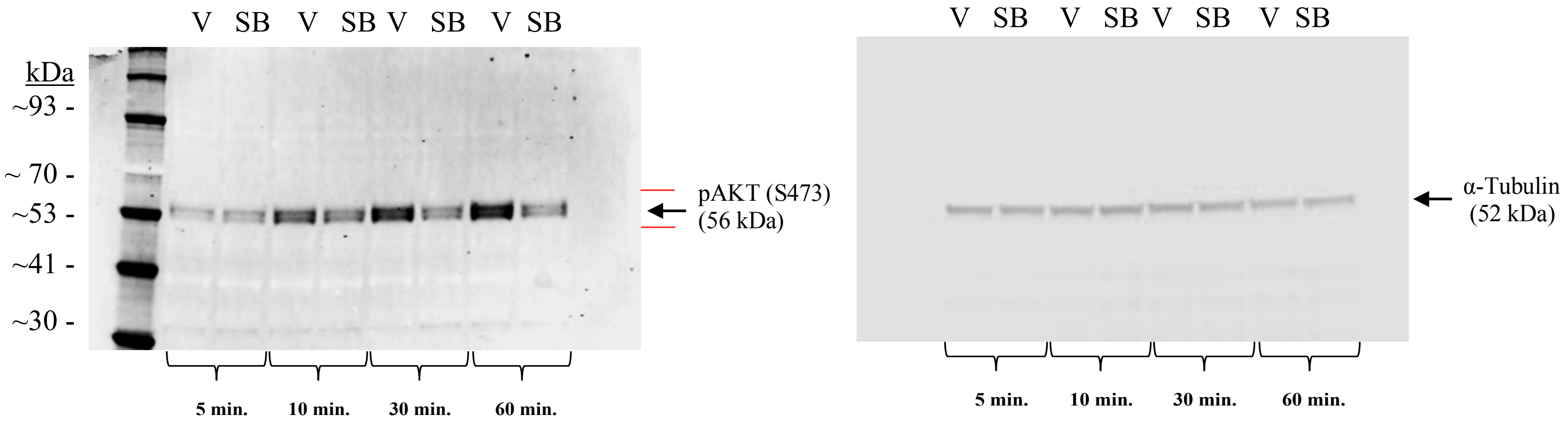

Blot 11

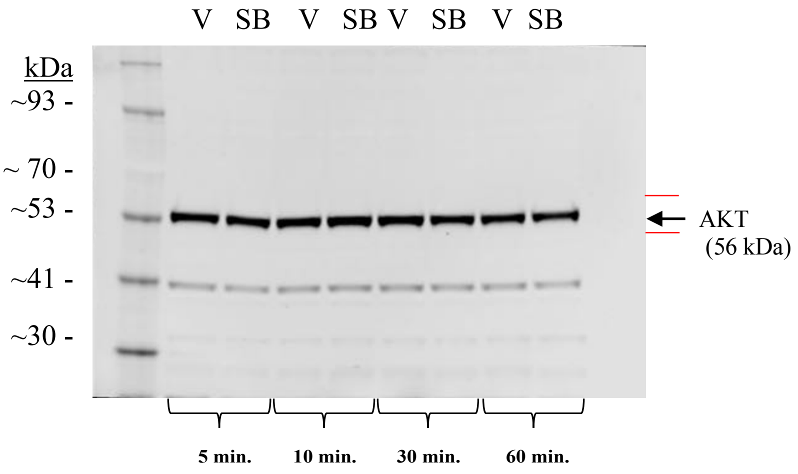

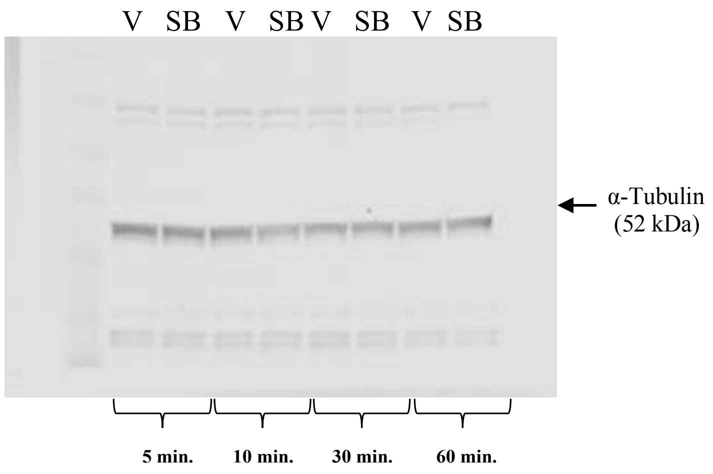

Blot 12

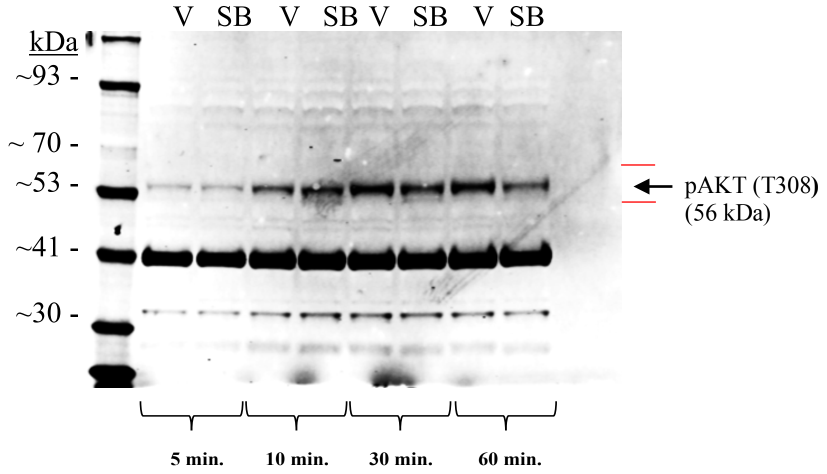

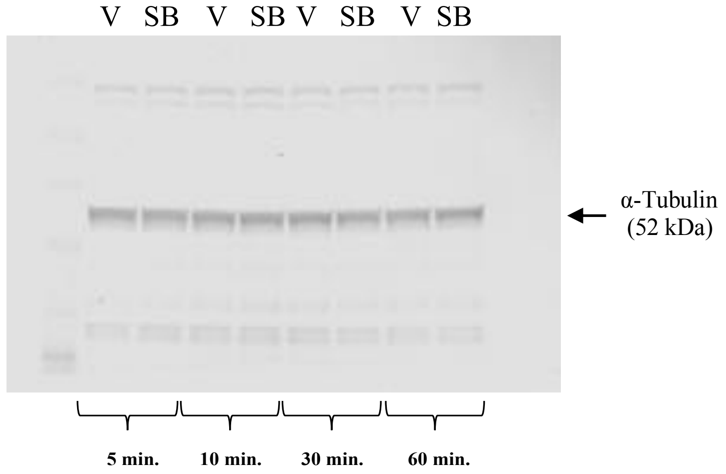

Blot13

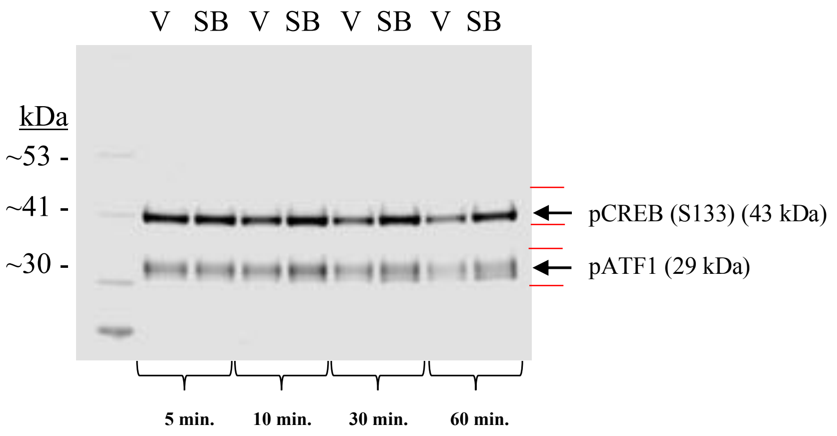

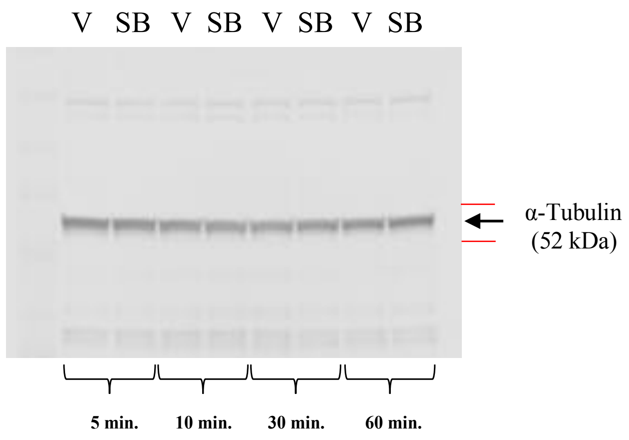

Blot14

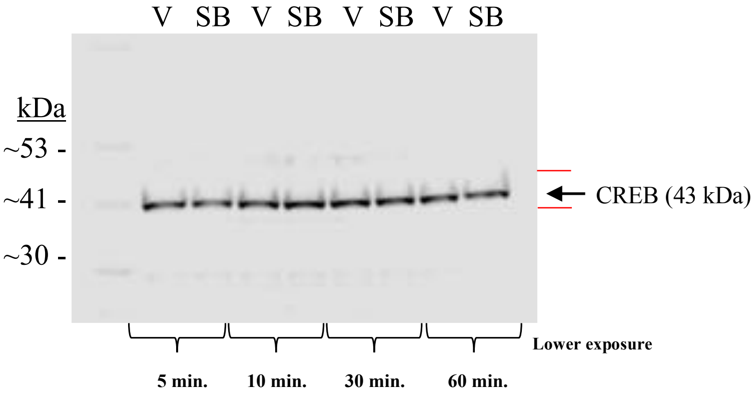

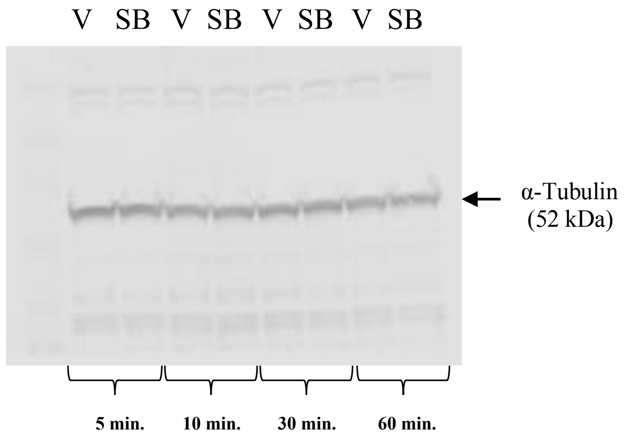

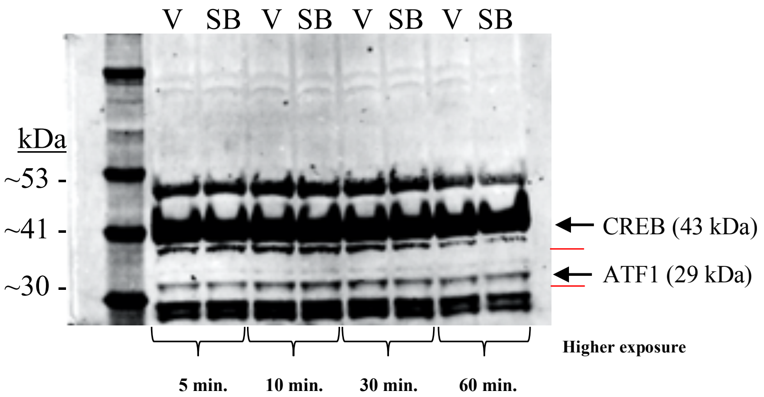

Blot15

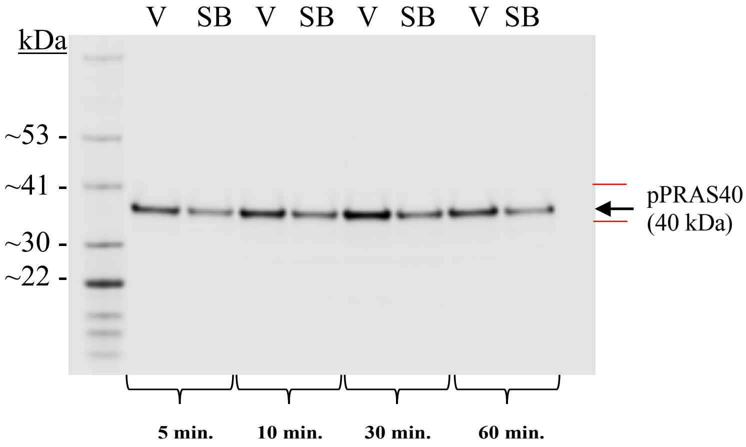

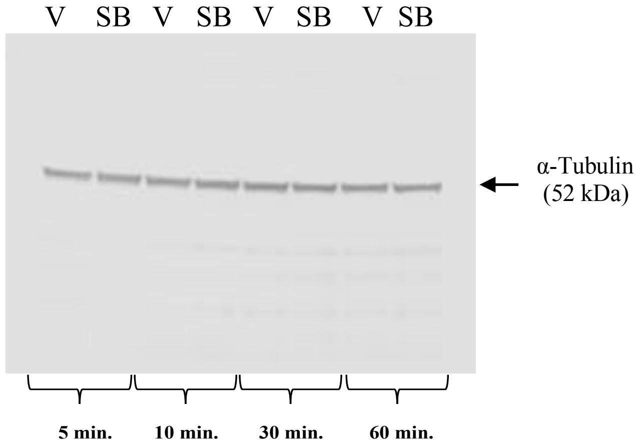

Blot16

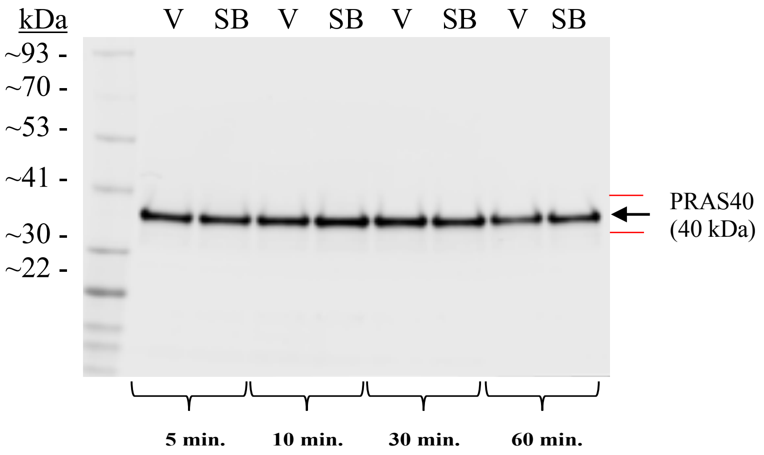

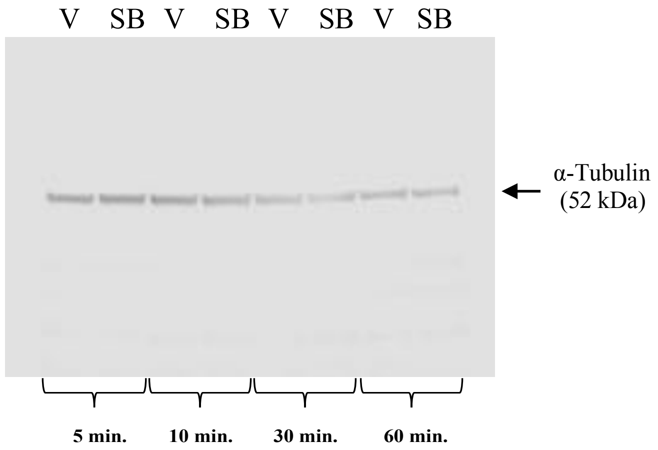

Blot17

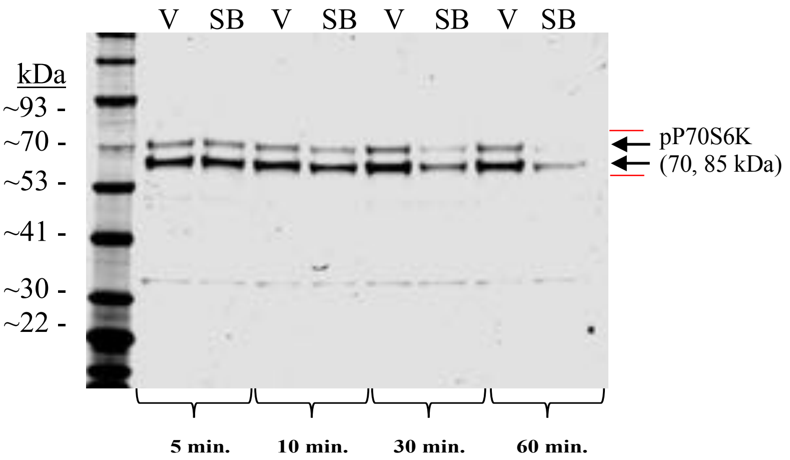

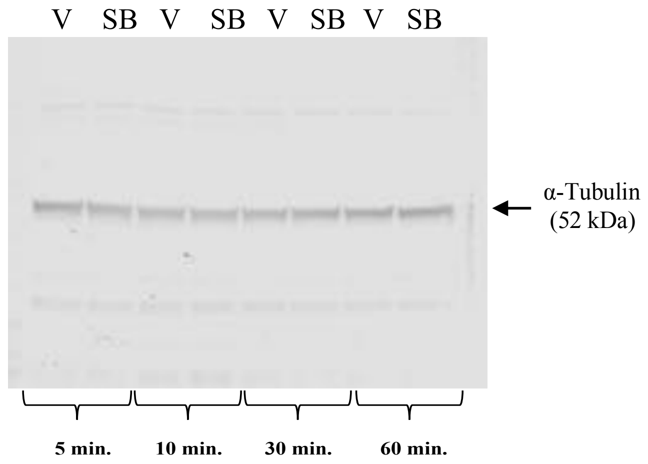

Blot18

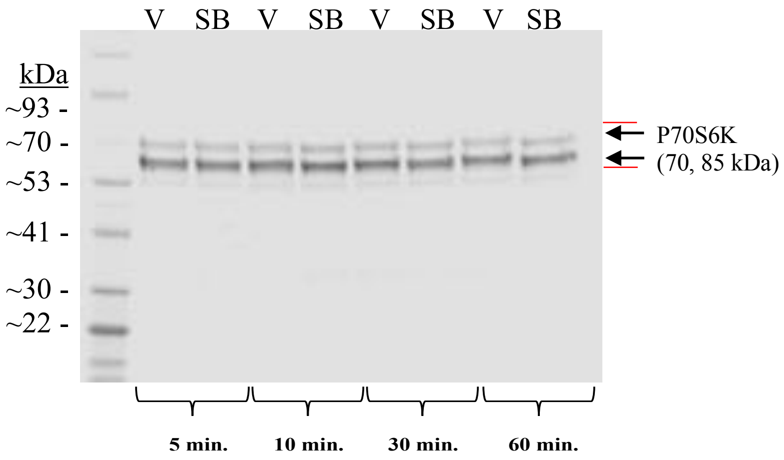

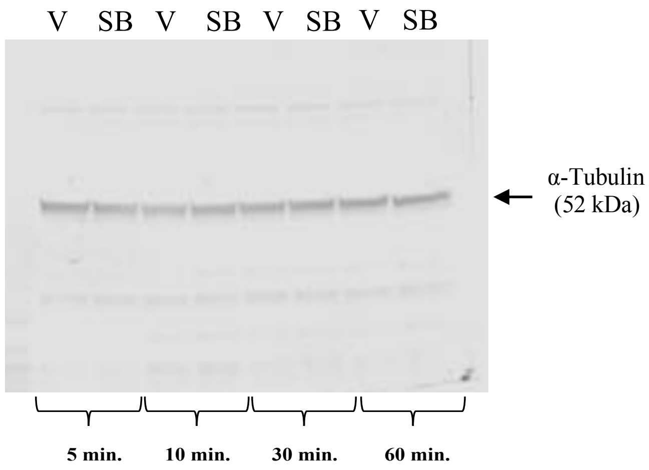

MCF-7 Figure 3F-G

Blot19

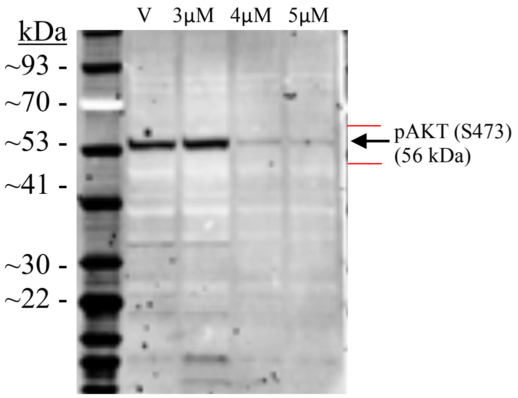

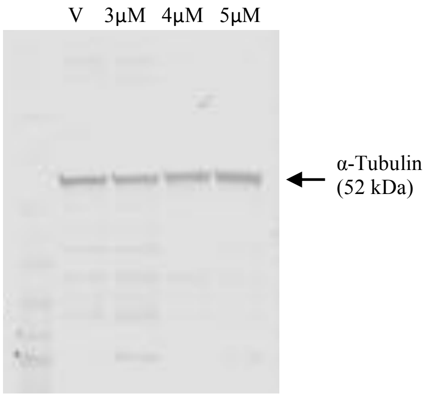

Blot20

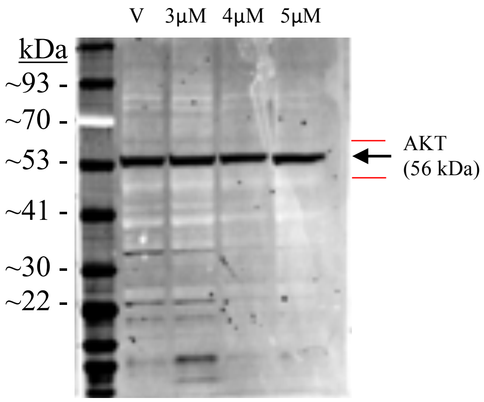

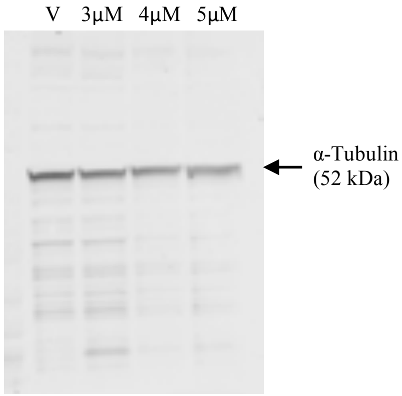

Blot21

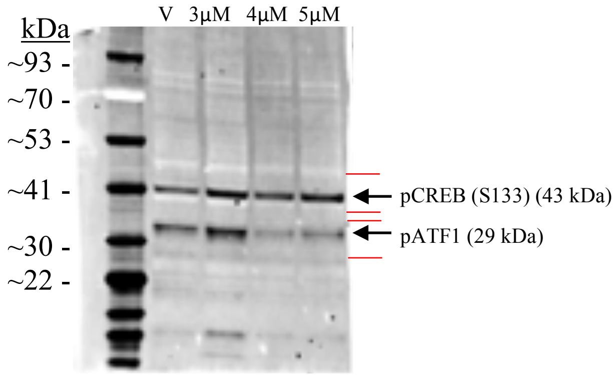

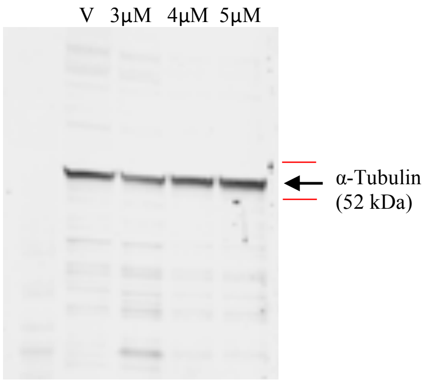

Blot22

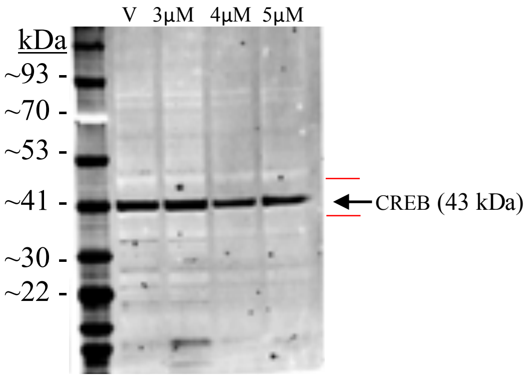

Lower exposure

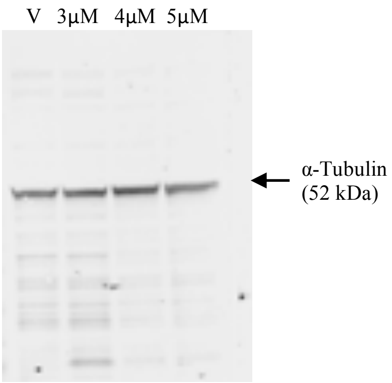

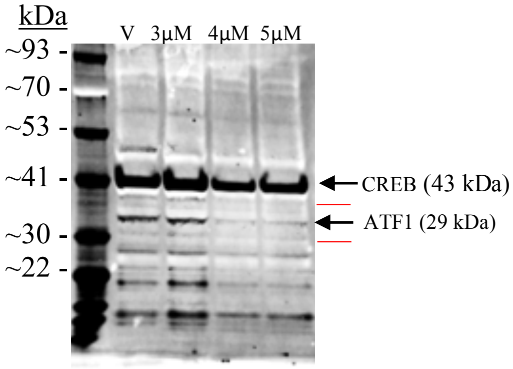

Higher exposure

Blot23

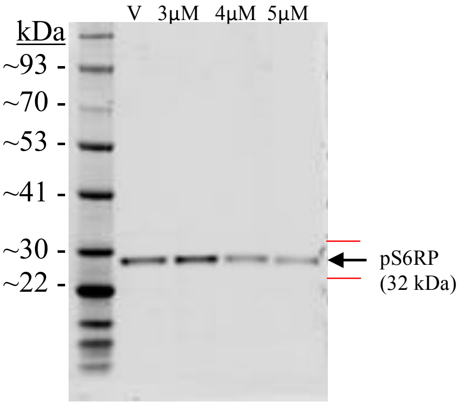

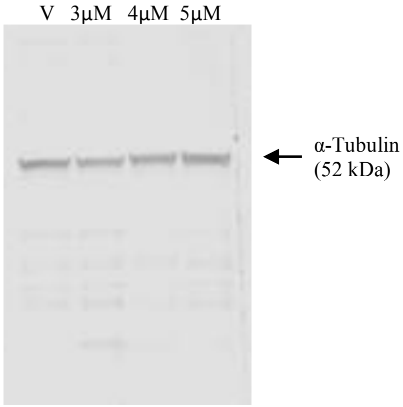

Blot24

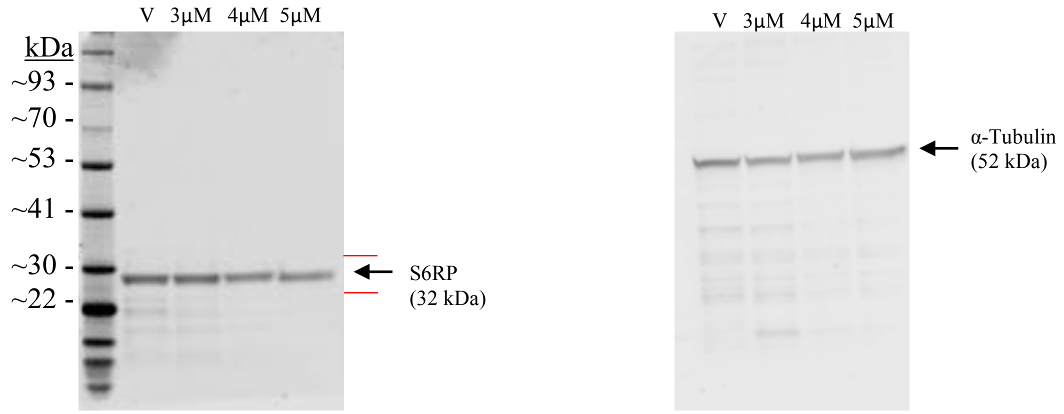

Blot25

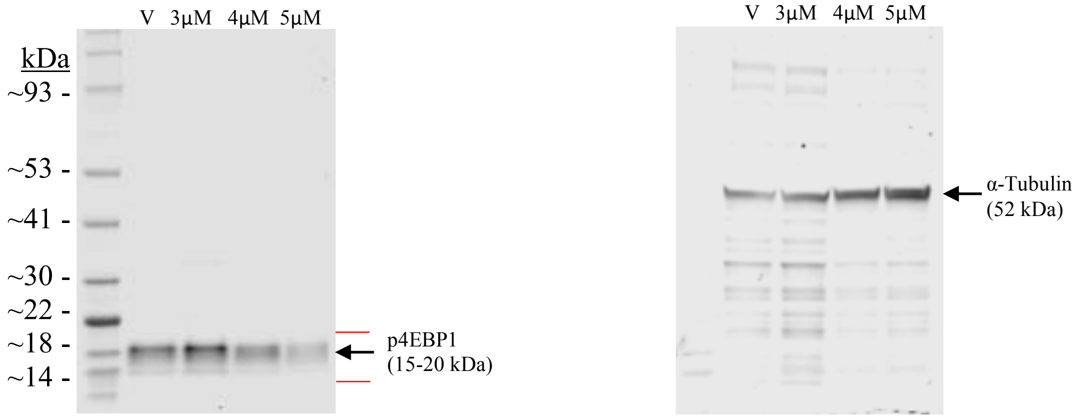

Blot26

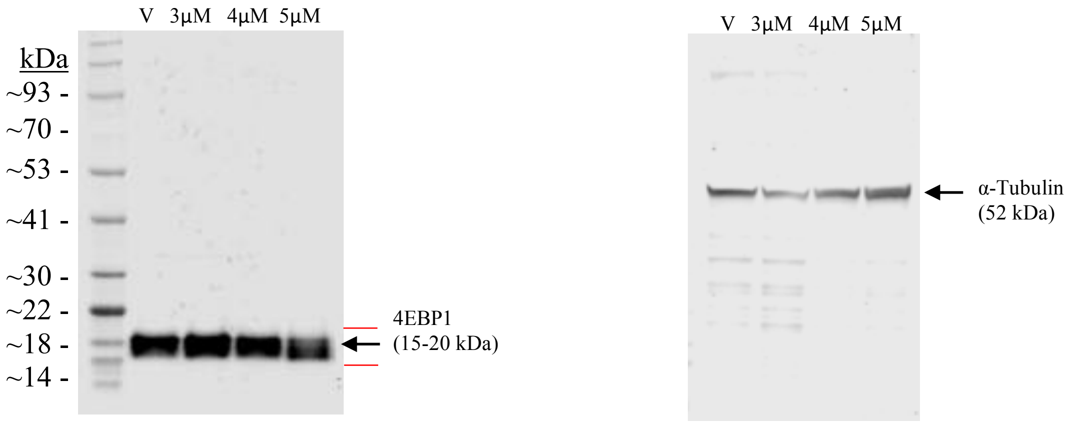

Blot27

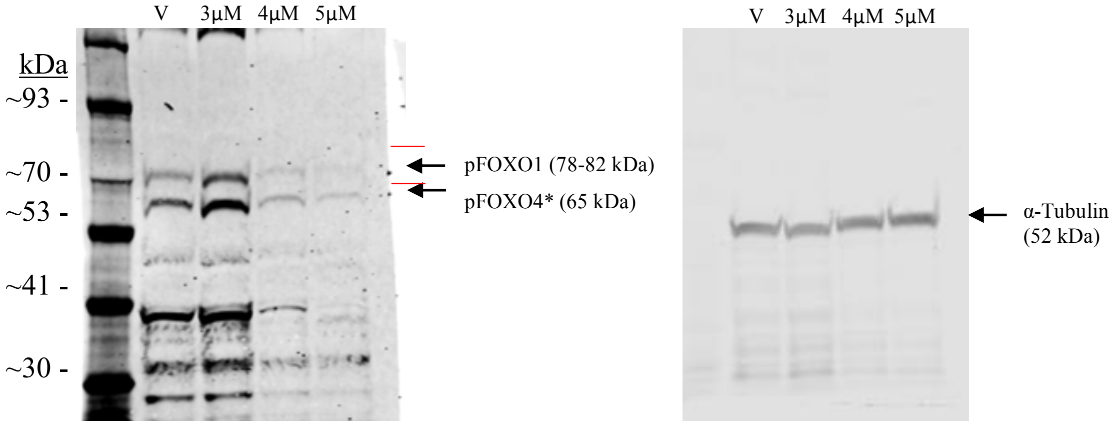

*antibody also recognizes pFOXO4,
which runs slightly below pFOXO1

Blot28

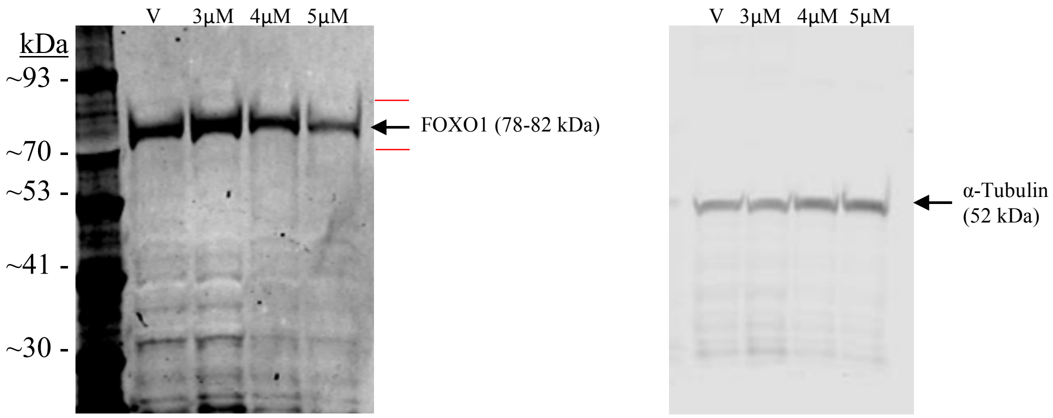

MDA-MB-157 Figure 3F-G

Blot29

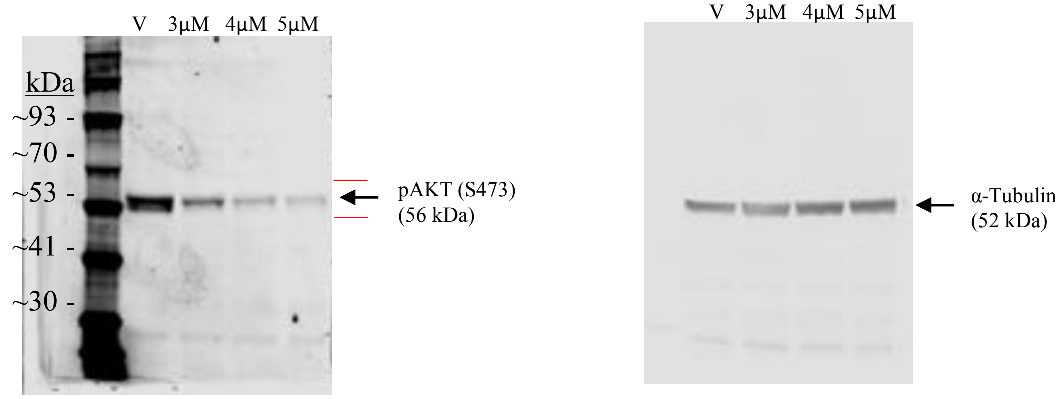

Blot30

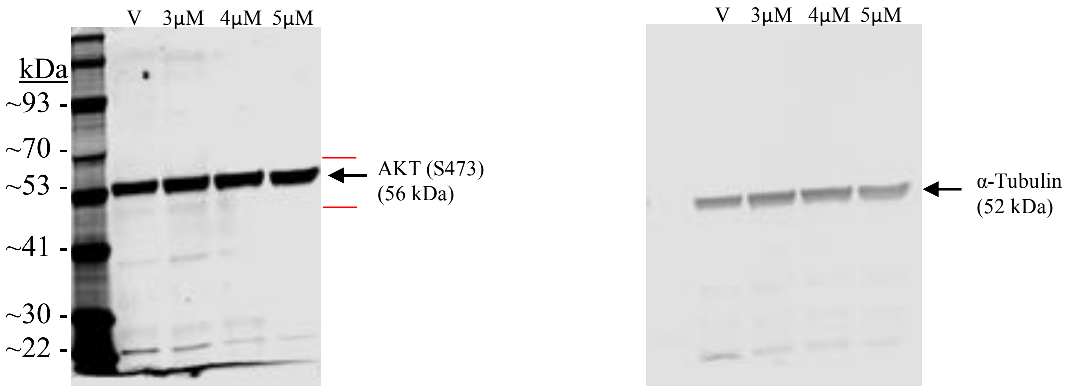

Blot31

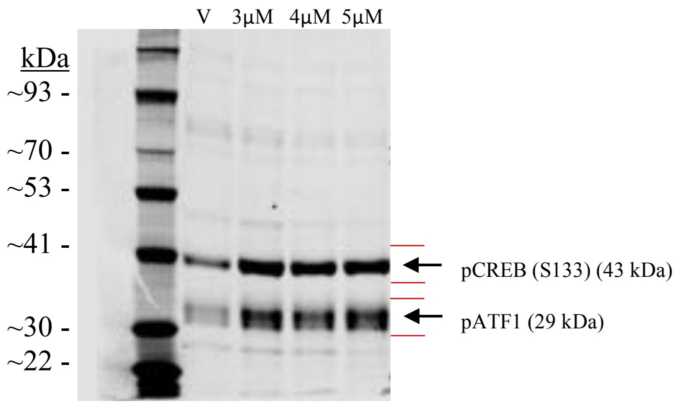

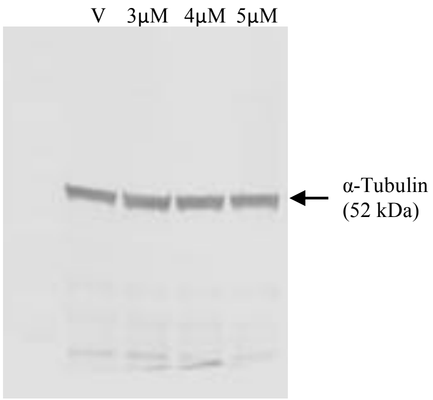

Blot32

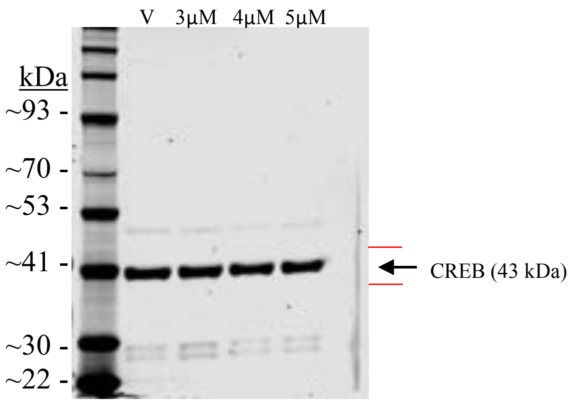

Lower exposure

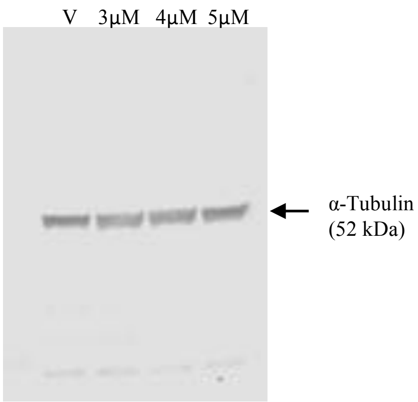

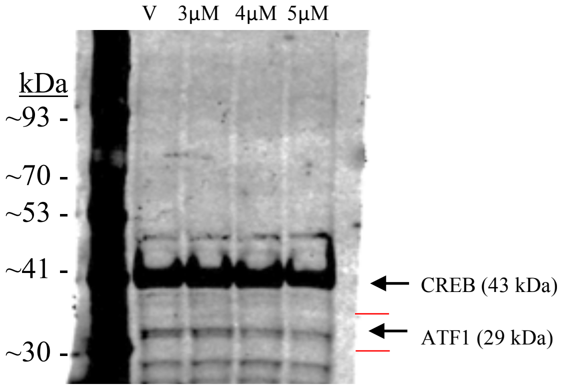

Higher exposure

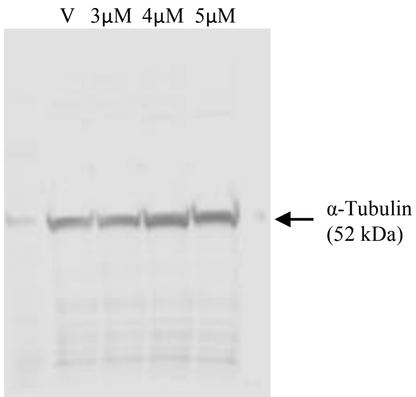

Blot33

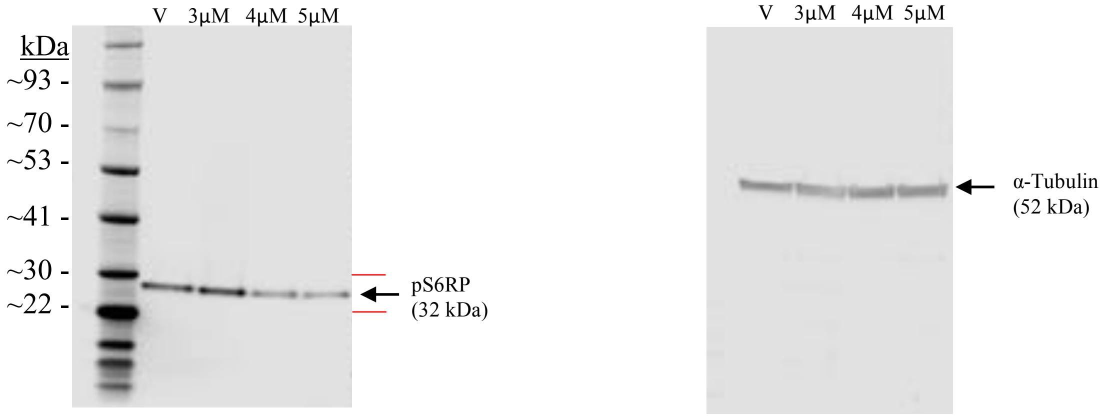

Blot34

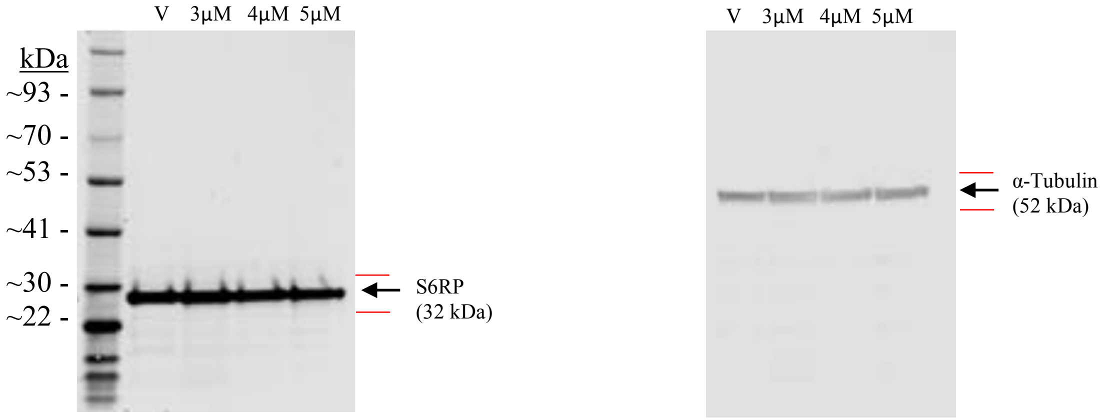

Blot35

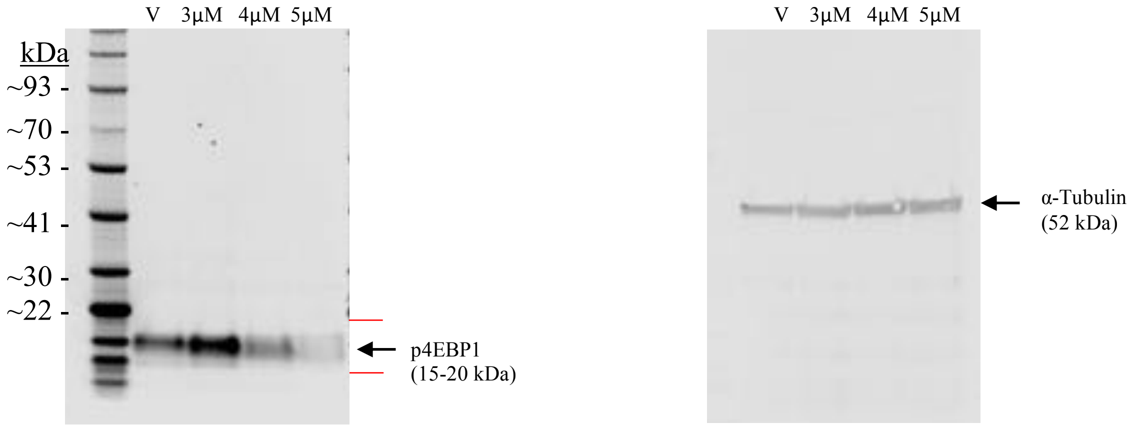

Blot36

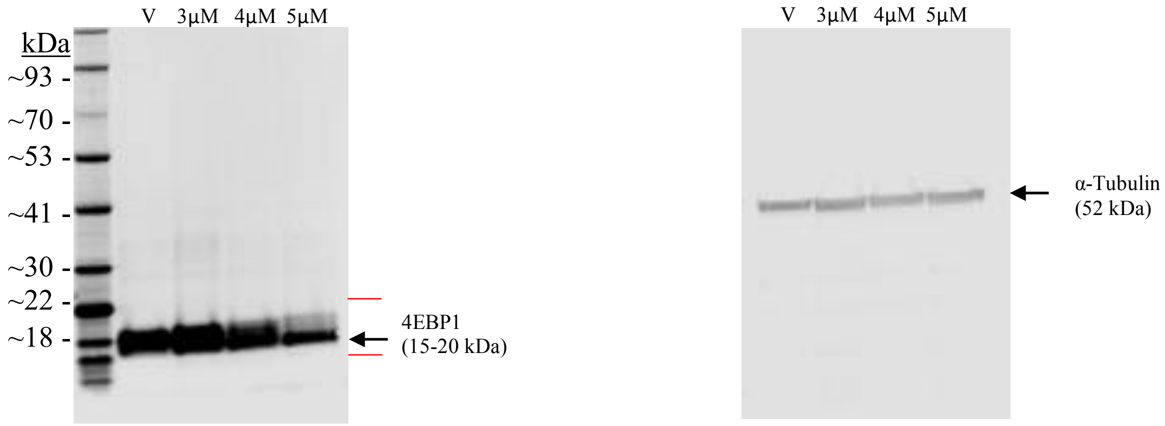

Blot37

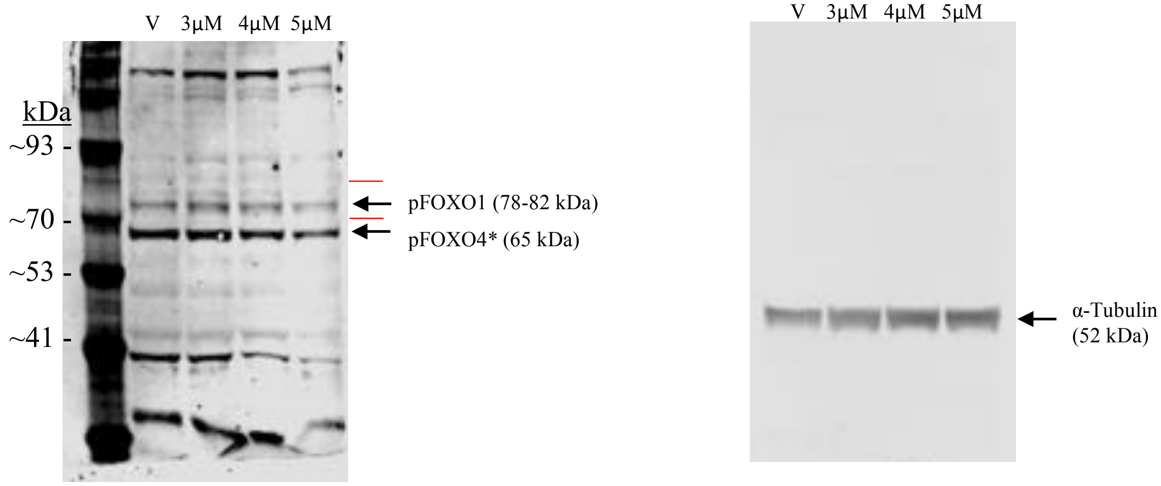

*antibody also recognizes pFOXO4, which runs slightly below pFOXO1

Blot38

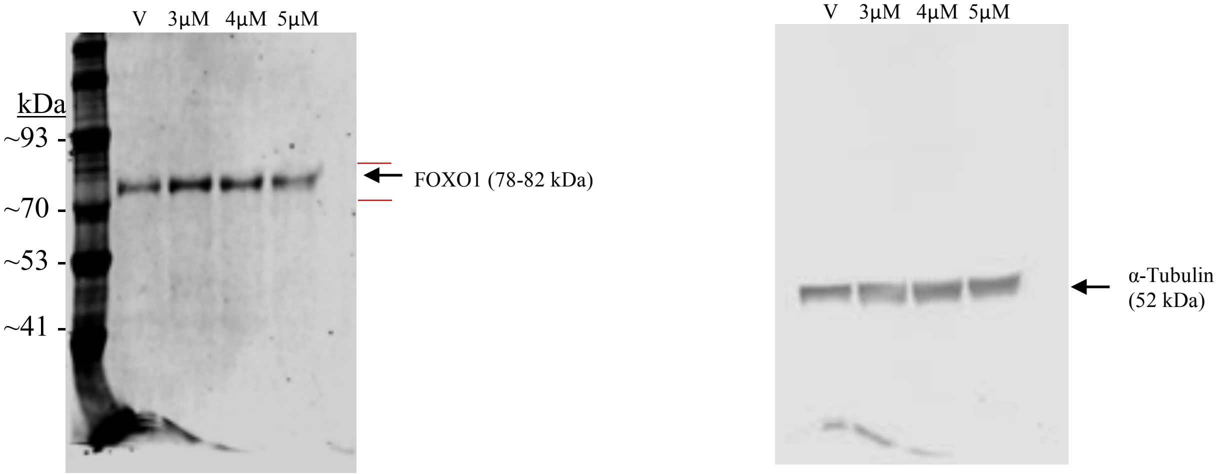

Supplement: Supplementary file 10 — Additional file 10: Figure S8. Uncropped western blots used in Fig. 3. Blots were imaged using the LI-COR Biosciences Odyssey Platform. Each blot was imaged under the 700 nM channel (left), which displays the molecular weight markers and the protein of interest, and under the 800 nM channel (right), which displays the α-Tubulin loading control. Blots were cropped where indicated by the horizontal red lines. [file 12885_2020_7193_MOESM10_ESM.pdf]
