# Supplementary material for: Antagonists of the serotonin receptor 5A target human breast tumor initiating cells
Source: BMC Cancer. 2020 Aug 5;20:724. doi: 10.1186/s12885-020-07193-6 (PMC7404930; doi:10.1186/s12885-020-07193-6)
Supplement: Supplementary file 11 — Additional file 11. Supplementary Methods. [file 12885_2020_7193_MOESM11_ESM.docx]

| **Experiment** | **Number of Mice** |
| --- | --- |
| *Ex vivo* experiment with tumor cells exposed to SB-699551 | 40 mice total: 10 transplanted with vehicle treated tumor cells  6 transplanted with tumor cells exposed to 300 µM SB-699551  10 transplanted with tumor cells exposed to 400 µM SB-699551  10 transplanted with tumor cells exposed to 500 µM SB-699551  4 transplanted with tumor cells exposed to 600 µM SB-699551 |
| *Ex vivo* experiments with *HTR5A iKO* | 32 mice total:  4 mice transplanted with 10,000 viable cells of dox-induced non-targeting iKO  4 mice transplanted with 10,000 viable cells of uninduced non-targeting iKO  4 mice transplanted with 10,000 viable cells of dox-induced *HTR5A* iKO 2-8  4 mice transplanted with 10,000 viable cells of uninduced *HTR5A* iKO 2-8  4 mice transplanted with 25,000 viable cells of dox-induced non-targeting iKO  4 mice transplanted with 25,000 viable cells of uninduced non-targeting iKO  4 mice transplanted with 25,000 viable cells of dox-induced *HTR5A* iKO 2-8  4 mice transplanted with 25,000 viable cells of uninduced *HTR5A* iKO 2-8 |
| *In vivo* maximum tolerable dose of SB-699551 | 10 mice total:  2 mice received docetaxel alone (10 mg/kg; intraperitoneally(i.p.))  8 mice received docetaxel (10 mg/kg) in combination with each of 12.5, 25, 37.5 and 40 mg/kg SB-699551 |
| *In vivo* preclinical study of SB-699551 efficacy | 62 mice total:  13 mice treated with the vehicle  13 mice treated with docetaxel only (10 mg/kg; intraperitoneally (i.p.))  18 mice treated with SB-699551 (25 mg/kg; intraperitoneally (i.p.))  18 mice treated with SB-699551 and docetaxel in combination (i.p.) |
